# Excitation energy dependence of ultrafast Jahn–Teller switching in Mn(iii) acetylacetonate

**DOI:** 10.1039/d6cp02713j

**Published:** 2026-08-26

**Authors:** Binoy Paine, Julien Eng, Kyle Barlow, Thomas J. Penfold, J. Olof Johansson, Alice E. Green, Ryan Phelps

**Affiliations:** a EaStCHEM School of Chemistry, University of Edinburgh David Brewster Road EH9 3FJ Edinburgh UK; b Chemistry, School of Natural and Environmental Sciences, Newcastle University Newcastle upon Tyne UK; c STFC Central Laser Facility, Research Complex at Harwell, Rutherford Appleton Laboratory, Harwell Campus Didcot OX11 0QX UK ryan.phelps@stfc.ac.uk

## Abstract

The photoinduced Jahn–Teller (JT) switch in Mn(acac)_3_ provides a model for how ultrafast structural dynamics can be harnessed to control magnetic anisotropy in single-molecule magnets (SMMs). However, the role of excitation energy in governing the efficiency and pathway of this transformation remains unclear. Here, ultrafast transient absorption spectroscopy (380–940 nm) probes the excitation-energy dependence of the JT switch. Selective excitation of Q_1_, Q_2_, and Q_3,4_ states shows that all pathways converge to a long-lived compressed-state photoproduct, but with distinct relaxation timescales, vibrational dynamics, and yields. Vibrational coherence analysis reveals modes at 170, 208, and 254 cm^−1^, assigned to the elongated, Q_1_ excited, and compressed state configurations, respectively. The 208 cm^−1^ mode is observed only under direct Q_1_ excitation, indicating excitation-dependent access to the JT-reactive coordinate. The compressed-state yield exhibits a strong dependence on excitation energy. For excitation into the lowest Q_1_ state (940–700 nm), the yield increases and follows a one-dimensional Landau–Zener model, where higher wavepacket velocity along the JT coordinate enhances nonadiabatic transition probability. The yield plateaus between 700–640 nm as the Q_2_ state is accessed and excess energy is likely transferred into non-reactive modes. Upon excitation into the Q_3,4_ manifold, the yield increases sharply, reaching a maximum near 470 nm before decreasing at higher energies, revealing the onset of multidimensional dynamics and competing relaxation pathways. These results identify excitation energy as a control parameter for the nonadiabatic structural dynamics, providing design rules for optically steering magnetic anisotropy in SMMs.

## Abstract 2

Landau–Zener dynamics govern the photoinduced Jahn–Teller switch in Mn(acac)_3_. By tuning excitation energy, the probability of nonadiabatic transfer and compressed-state formation can be controlled, offering a route to steer magnetic anisotropy.

## Graphical abstract

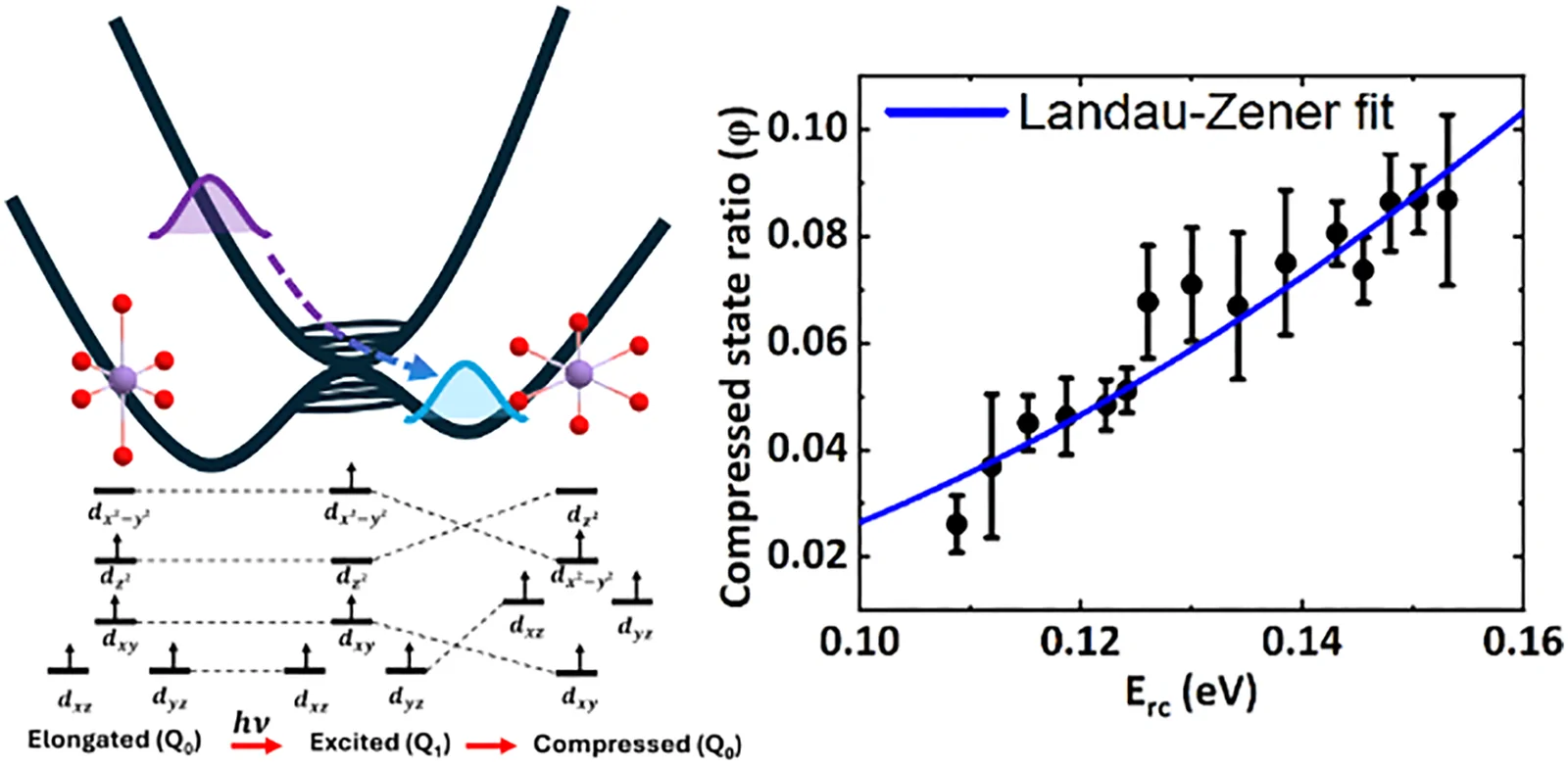

## 1. Introduction

The Born–Oppenheimer (BO) approximation is fundamental to the study of molecular dynamics, enabling the separation of electronic and nuclear motion and the construction of adiabatic potential energy surfaces (PESs). However, in polyatomic molecules, this framework fails near conical intersections (CIs),^1,2^ where electronic states become close in energy and strongly couple to nuclear motion. At these seams, nonadiabatic coupling can dominate the dynamics, allowing ultrafast population transfer between PESs on timescales of tens to hundreds of femtoseconds, preceding substantial thermal equilibration.^3–5^ Consequently, the topography of these intersections and the velocity of the nuclear wavepacket often play decisive roles in guiding excited state reactivity.^6,7^

Within the Landau–Zener (LZ) framework, the probability (*P*) of a nonadiabatic transition at a given nuclear coordinate is expressed as eqn (1) and is determined by the velocity (*v*) of the wavepacket, the coupling strength (*V*_12_), and the difference in the gradients (Δ*F*) of the potential energy surfaces.^8^1
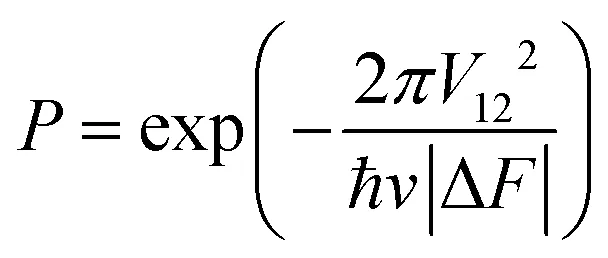
Specifically, the probability of a nonadiabatic transition increases with higher velocities and steeper gradient differences, while it decreases as the nonadiabatic coupling strength increases, as the latter widens the adiabatic energy gap. Consequently, methods which control the initial velocity of the wavepackets along reactive modes offer a potential handle for steering reactivity. This principle has been established in organic photochemistry,^9^ for example, in the *cis*–*trans* isomerisation of retinal, where the direct observation of vibrational coherences explicitly confirmed the presence of wavepacket dynamics along the reactive torsional mode.^10^ Adjusting the excitation wavelength from 570 nm to 510 nm deposits greater vibrational energy along this coordinate, generating a faster wavepacket that increased reaction yield by 5%.^11,12^ This quantum yield plateaus at lower excitation wavelengths (450–500 nm). At these higher photon energies, excess energy is preferentially partitioned into photochemically inactive, high-frequency modes rather than the reactive torsional coordinate, preventing any further increase in wavepacket velocity. Conversely, wavepackets with minimal excess energy (>570 nm) get trapped on the initial surface.

Ultrafast optical and X-ray spectroscopic studies of transition metal complexes have identified a range of wavepacket dynamics that can guide a system toward conical intersections, including ligand pincer motions and metal-centred distortions,^13–17^ such as metal–ligand stretching, axial or equatorial breathing, and symmetry-breaking motions. Selective excitation of specific modes can alter the accessible regions of the PES, thereby steering the system along different reaction pathways.^18^ For example, in Fe(ii) spin crossover complexes, the ultrafast intersystem crossing (ISC) rates are governed by the density of accessible electronic states and vibronic coupling to Fe–N stretching and metal-centred breathing coordinates.^19,20^ The population cascades rapidly through the singlet–triplet–quintet manifold along metal-centred breathing and Fe–N stretching modes, with the overall ISC completing within 150 fs.^21–23^ In Re(i) carbonyls, despite the heavy-atom effect providing substantial spin–orbit coupling, ISC rates were found to scale inversely with the spin–orbit coupling constant of the halide ligand, demonstrating that coupling strength alone does not determine the transition rate.^24^ Instead, a strong correlation was found between the ISC timescale and the vibrational period of the Re–L skeletal mode, indicating that low-frequency structural motions of the Re(L)(CO)_3_ unit provide the vibronic coupling necessary to drive a rapid, nonadiabatic transition between the singlet and triplet manifolds. Despite extensive efforts to understand these ultrafast transformations, the influence of photon energy on ultrafast nonadiabatic transitions remains largely unexplored.

**Fig. 1:**
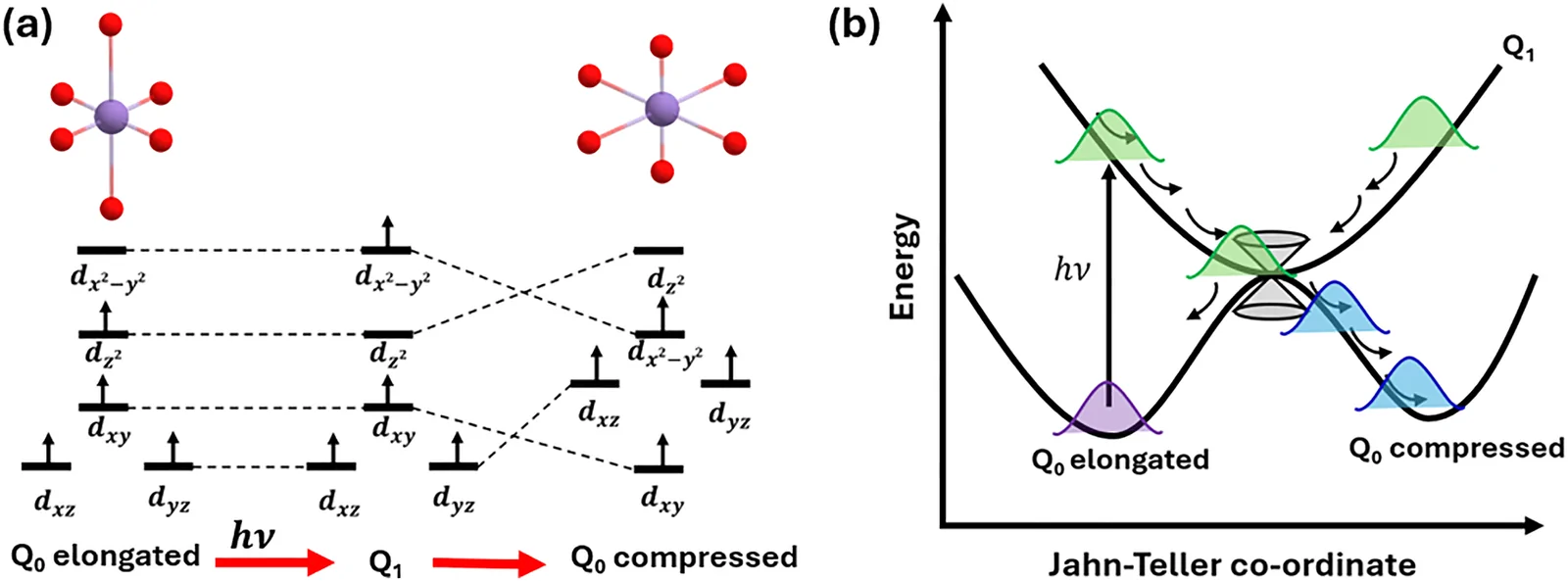
(a) Schematic of the photo-induced Jahn–Teller (JT) switching mechanism in Mn(iii) complexes. (b) Schematic potential energy curves of the electronic states involved and the corresponding wavepacket dynamics during JT switching. Photoexcitation generates a wavepacket on the Q_1_ state (green), which can then return to the Q_0_ compressed (blue) and elongated (purple) states. The arrows illustrate JT-motion after excitation, forming the compressed state during the first passage through the conical intersection or the elongated state on return of the wavepacket to the conical intersection.

Herein, we focus on the dynamics of the photoinduced Jahn–Teller (JT) switch in Mn(iii) complexes, as illustrated in Fig. 1, which has emerged as a promising mechanism for manipulating the magnetic anisotropy of single-molecule magnets (SMMs).^13,25^ This behaviour arises from the high-spin d^4^ electronic configuration of Mn(iii) 
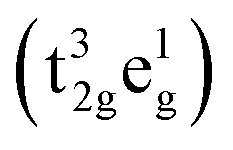
, which leads to a strong coupling between electronic structure and molecular geometry *via* the JT effect. As a result, changes in coordination geometry are intrinsically linked to the magnetic anisotropy in these systems. The ability to control such distortions with light is particularly attractive because it offers a route to the ultrafast manipulation of magnetic properties,^26^ with potential applications in molecular spintronics,^27^ high-density information storage,^28^ and quantum information technologies.^29^ Consequently, understanding the structural dynamics that govern JT switching is central to the rational design of next-generation photoresponsive molecular magnetic materials.^30^ Johansson and co-workers previously explored the vibrational dynamics underlying JT switching in model Mn(iii) complexes, seeking to identify the key reaction coordinates that control this process on ultrafast timescales.^13–16,31^ In their studies, ultrafast transient absorption spectroscopy (TAS) was employed to probe wavepacket dynamics following 400 nm photoexcitation of both a Mn_3_ SMM and the model complex Mn(acac)_3_ (acacH = acetylacetone).^13^ Analysis of oscillatory features superimposed on the kinetic TAS traces revealed vibrational coherences associated with the ultrafast photoinduced switching between elongated and compressed JT distortions. This work was later extended to the bulkier derivative tris(2,2,6,6-tetramethyl-3,5-heptanedionato) manganese(iii), Mn(dpm)_3_, slowing the dynamics and enabling direct tracking of the excited-state vibrational wavepacket as it evolved toward and through a conical intersection.^16^ Passage through this region partitioned the coherence between the elongated and compressed JT minima (blue and purple in Fig. 1 (b)). By monitoring the relative phase of the resulting wavepackets, a phase-resolved picture of the switching process was obtained, providing direct insight into the momentum flow that drives JT reorganisation. This work thereby established a mechanistic foundation for future strategies aimed at manipulating the structural dynamics of single-molecule magnets on ultrafast timescales.

The present study builds on this foundation by using ultrafast TAS to map the excitation energy dependence of the photoinduced JT switch in Mn(acac)_3_, enabling direct interrogation of how excess energy deposition influences wavepacket trajectories and switching efficiency. This work identifies excitation energy as a powerful experimental handle for directing nonadiabatic structural transformations, with implications for the rational control of photo-responsive SMMs.

## 2. Method

Samples of Mn(acac)_3_ (Sigma-Aldrich, 97%) were prepared in both ethanol and dichloromethane (Sigma-Aldrich, HPLC grade). Steady-state and temperature-dependent UV-Visible absorption spectra were collected using a Shimadzu UV-1800 spectrophotometer. Measurements of 5 mM dichloromethane solution were acquired between 15 and 35 °C using a 1 cm quartz cuvette. Dichloromethane was selected as an aprotic, non-hydrogen-bonding solvent to maintain a constant solvation environment, thereby avoiding the temperature-induced disruption of hydrogen-bond networks and subsequent changes in effective polarity observed in protic solvents like ethanol, ensuring that any spectral shifts reflect the intrinsic behaviour of the complex (see SI Section S1 for expanded discussion and comparative data).

The excitation energy dependent nonadiabatic dynamics of ∼50 mM Mn(acac)_3_ solutions in ethanol and dichloromethane (spectra shown in Fig. S4 of the SI) were studied using TAS (the complete set shown in Fig. S11–S13 of the SI).^14–16^ Ethanol solutions were excited with wavelengths ranging from 940 nm to 380 nm, and for comparison, two selected wavelengths (940 nm and 400 nm) were used for the dichloromethane solutions. Pump pulses with a pulse duration of 40–100 fs and spectral bandwidths of 5–35 nm (FWHM) were produced from a non-collinear optical parametric amplifier (ORPHEUS-F, Light Conversion) pumped by the 1030 nm output from a Yb: KGW regenerative amplifier (PHAROS-PH2) running at 1 kHz with a pulse duration of 270 fs. A weak broadband white-light continuum probe was generated by focusing (*f* = −10 cm) the horizontally polarised 1030 nm fundamental into a 5 mm thick CaF_2_ plate, which was rastered to avoid degradation. Prior to focusing in the CaF_2_ plate, the 1030 nm fundamental was spatially filtered using a 4.8 mm iris. The pulse energy of the 1030 nm was 3 µJ prior to this filtering, and 2.5 µJ post filtering.

The sample solutions were flowed at 16 µL min^−1^ through a Starna quartz flow cell with a 0.2 mm path length. Although this results in the same volume of sample being irradiated multiple times while traversing the interaction region, any excited-state population generated by a laser pulse relaxes well before subsequent excitation events. The vast majority of the excited-state population recovers on the picosecond timescale, while minor long-lived signals persist only into the nanosecond regime. Consistent with this, no signal was observed at negative time delays, indicating the absence of residual excited-state species or accumulated photoproducts. The fluence of the pump pulses ranged from 0.58 to 4.63 mJ cm^−2^ depending on the energy available for a particular wavelength. Fluence-dependent measurements carried out across this range confirmed a strictly linear signal response, ensuring that the probed dynamics are free from multiphoton processes (see Section S2 of the SI). A temporal cross-correlation of ≤180 fs across the probe spectral range was achieved in pure ethanol and dichloromethane. The relative polarisation of the pump and probe pulses was set to the magic angle (54.7°). A motorised optical delay line in the pump path controlled the delay between the pump and probe pulses. A mechanical chopper running at 500 Hz blocked alternating pump pulses to produce pump-on and pump-off spectra, the combination of which gives transient absorption difference spectra at each time delay. After the sample stage, the probe was collimated and dispersed by a prism set at the Brewster angle onto a fast charge-coupled device (CCD) camera (Entwicklungsbuero Stresing) equipped with a Hamamatsu S7031-0907 sensor with 512 × 58 active pixels. A portion of the probe beam was split prior to the sample using a beamsplitter and passed to a matched detector to serve as a reference for each probe pulse.

## 3. Results and discussion

### 3.1. Electronic structure of Mn(acac)_3_

**Fig. 2:**
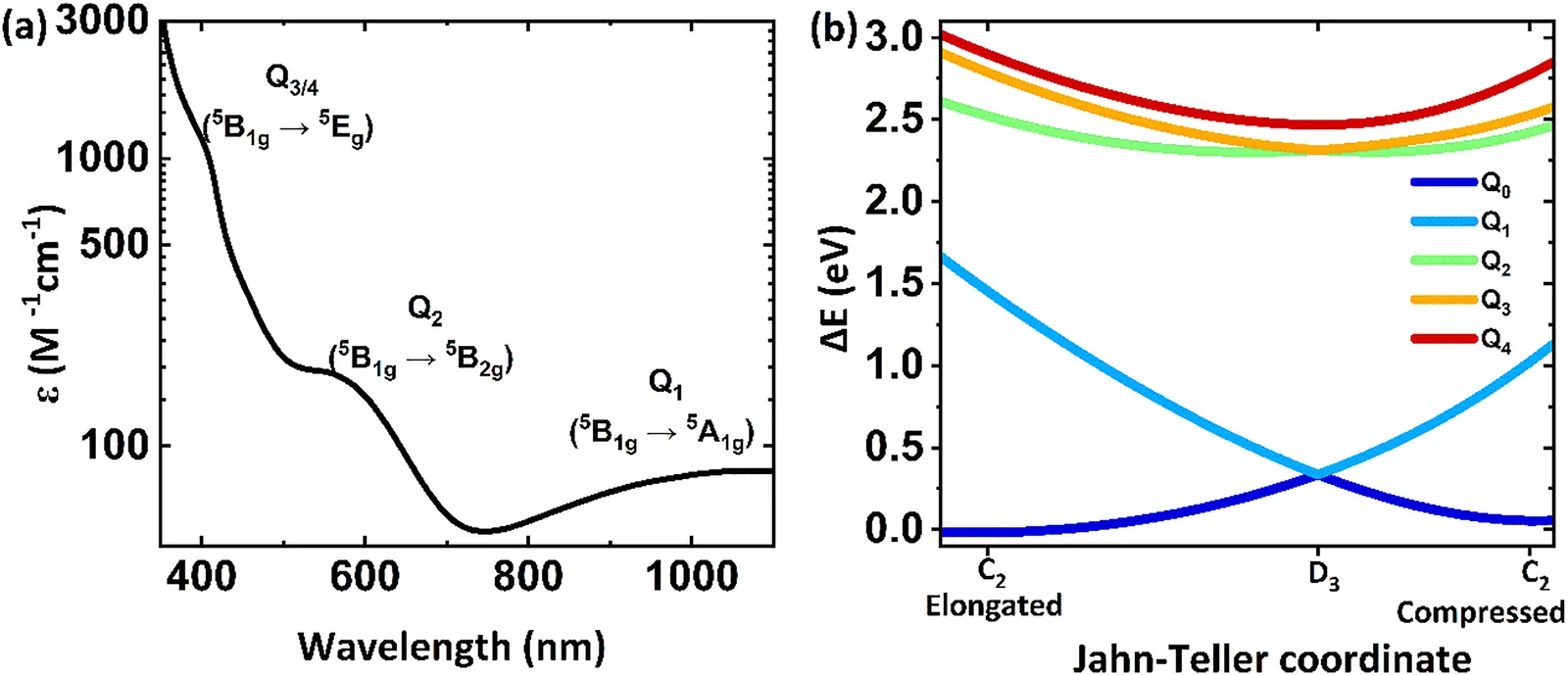
(a) UV-Visible absorption spectrum of Mn(acac)_3_ in ethanol at room temperature recorded with a Shimadzu UV-1800 spectrophotometer, with intensity given on a logarithmic scale. (b) Computed PES scan of the first five quintet states along two pathways, calculated at the B3LYP/def2-TZVP level of theory using DFT/TD-DFT. The scan was performed along: (i) the linearly interpolated path in Cartesian coordinates between the elongated ground state minimum (*C*_2_) and the conical intersection (Q_1_ minimum), and (ii) the linearly extrapolated path in Cartesian coordinates between the Q_1_ minimum (*D*_3_) and the compressed minimum (*C*_2_).

The UV-Visible absorption spectrum of Mn(acac)_3_ in ethanol is shown in Fig. 2(a). Three broad, spin-allowed metal-centred transitions are observed originating from the ^5^B_1g_ ground state of the axially elongated JT geometry. The lowest energy feature, appearing at wavelengths above 700 nm, is assigned to the ^5^B_1g_ → ^5^A_1g_; Q_1_. A second band near 550 nm corresponds to the ^5^B_1g_ → ^5^B_2g_; Q_2_ transition, while the strong absorption around 400 nm arises from the higher-energy ^5^B_1g_ → ^5^E_g_; Q_3,4_ manifold. These assignments are consistent with the multireference CASSCF calculations reported in the SI Section S3 (Table S2) and are in good agreement with previously reported experimental and computational data.^13,32^Fig. 2(b) shows computed potential energy surfaces (PES) along the JT coordinate for the first five quintet states of Mn(acac)_3_. The lowest two states (Q_0_ and Q_1_) define the primary relaxation pathway, with Q_0_ corresponding to the ground-state surface and Q_1_ representing the first excited state. These two surfaces approach one another near the region of *D*_3_ symmetry (∼0.33 eV), where the energy gap becomes minimal, enabling strong coupling between them. This crossing region acts as a nonadiabatic funnel, facilitating rapid population transfer from Q_1_ back to Q_0_ along the JT-active distortion coordinate, which agrees with the short Q_1_ state lifetime observed previously and herein. In contrast, the higher excited states (Q_2_–Q_4_) form a dense but relatively shallow manifold that also converges in energy around the region of *D*_3_ symmetry. The small splitting between these surfaces indicates strong nonadiabatic coupling within the higher-lying states, allowing rapid population redistribution prior to decay into the lower states.

### 3.2. Temperature-dependent UV-Vis spectroscopy

**Fig. 3:**
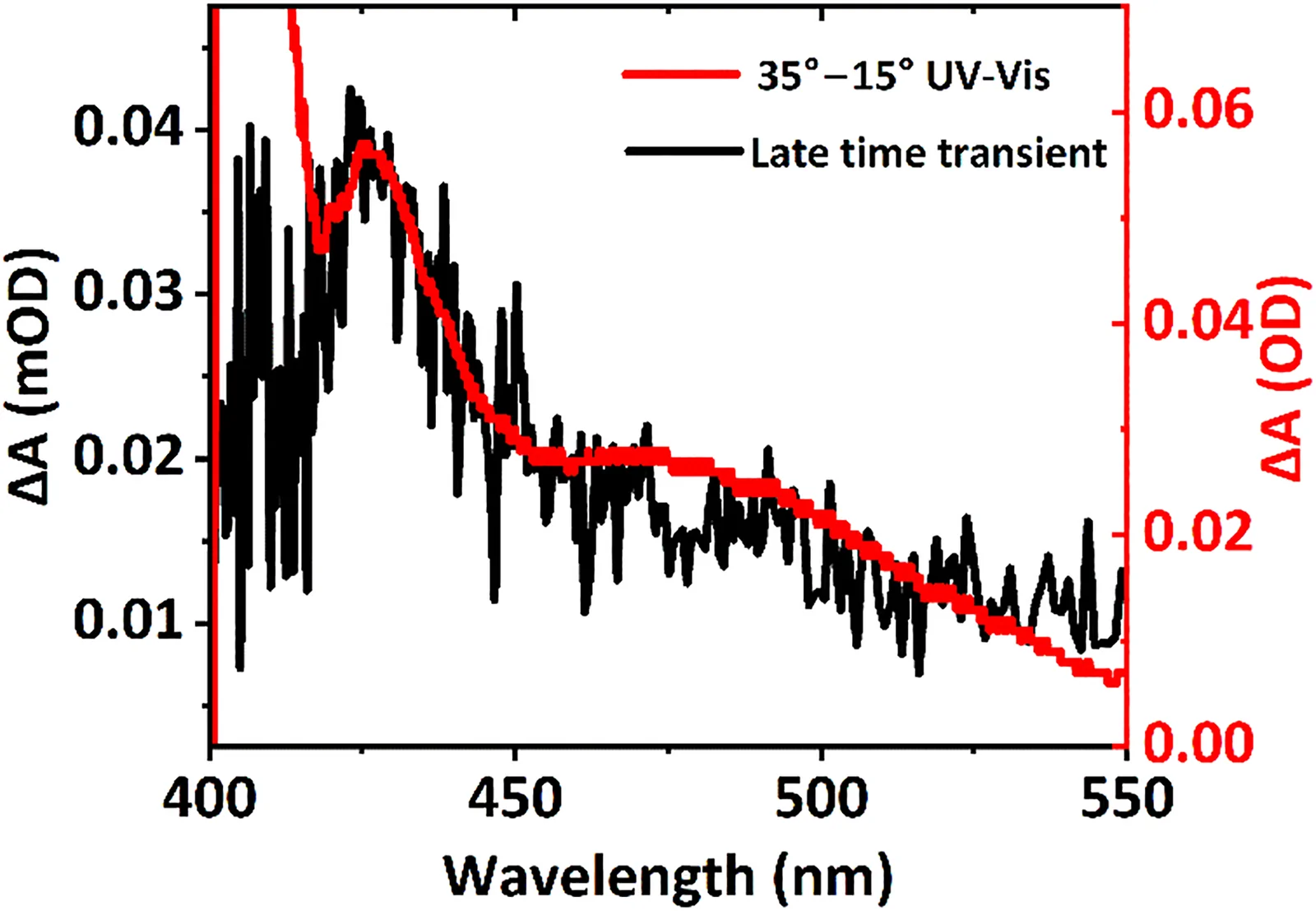
Comparison of the transient difference absorption spectrum (black) of 50 mM Mn(acac)_3_ in dichloromethane following photoexcitation at 940 nm, averaged over the 30–100 ps time window, with the difference spectrum (red) obtained from temperature-dependent UV-Vis spectroscopy (35–15 °C) of 5 mM Mn(acac)_3_ in dichloromethane.

The difference between the UV-Vis spectra recorded at 35–15 °C is shown in Fig. 3 and provides insight into the thermal accessibility of the compressed state. The difference spectrum between 35 °C and 15 °C reveals the emergence of a new absorption feature centred around 430 nm at elevated temperature. This indicates increased population of a higher-energy species whose absorption profile is red-shifted relative to the lowest-energy geometry. This thermally induced band closely matches the long-lived transient feature observed at 430 nm in the TAS measurements, as shown in Fig. 3, providing strong experimental evidence that both signals originate from the compressed-state configuration. The correspondence between the steady-state and time-resolved spectra supports assignment of the long-lived TA component to population trapped in this state. The relatively small energy separation between the elongated and compressed configurations, as suggested by our calculations (∼0.07 eV), is consistent with the observed thermal accessibility of this state. Minor differences between the thermal difference spectrum and the transient spectra in the 400 nm region are likely attributable to temperature-dependent changes in the extinction coefficient around the intense Q_3,4_ band where ligand-centred states also overlap, rather than to fundamental differences in the underlying electronic structure. Notably, despite thermal population of the compressed state occurring at the expense of the elongated ground-state conformer, no clear bleaching feature is observed in the difference spectrum. Instead, the spectrum is dominated by a positive band at 430 nm, suggesting that the compressed-state configuration possesses a substantially larger transition dipole moment, and therefore a greater oscillator strength than the elongated geometry.

### 3.3. Excitation wavelength dependence of ultrafast dynamics

Previous ultrafast studies of Mn(acac)_3_ have largely focused on excitation around 400 nm, where the intense Q_3,4_ band overlaps with higher-lying ligand-centred states, making it challenging to unambiguously determine which electronic levels participate in the JT switching mechanism.^13^ In the present work, we instead employ a range of excitation wavelengths (380–940 nm) to selectively prepare distinct excited states. This approach allows the dynamics to be interrogated from different initial conditions and enables a systematic assessment of how this influences the structural evolution and the overall JT switching mechanism. To capture these effects, the datasets are analysed in a series of complementary sections, focusing on spectral evolution, relaxation kinetics, coherent dynamics, and compressed-state yields, all of which help to build a comprehensive understanding of the underlying mechanism governing the photoinduced JT switch. Additional TAS datasets acquired using dichloromethane as the solvent are presented and discussed in Section S1 of the SI. Overall, the transient spectral evolution is highly similar in both solvents, indicating that the underlying excited-state dynamics are largely preserved. The principal difference is a greater relative intensity of the compressed-state features in DCM, although the sequence and nature of the spectral transformations remain unchanged.

#### 3.3.1. Spectral evolution

**Fig. 4:**
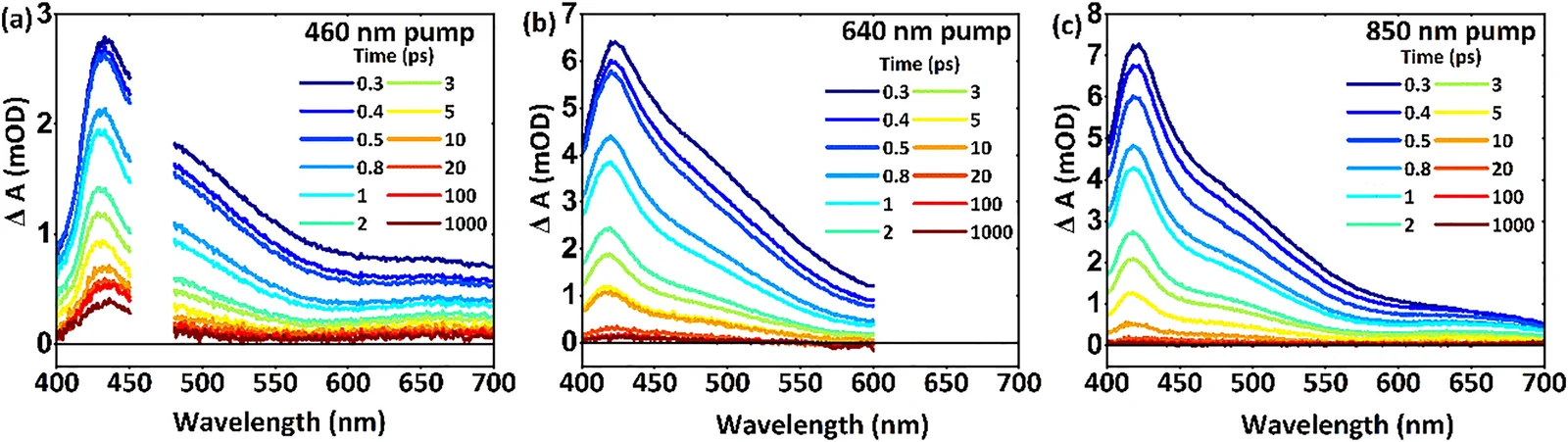
Transient absorption difference spectra of Mn(acac)_3_ in ethanol following photoexcitation at (a) 460 nm, (b) 640 nm, and (c) 850 nm, corresponding to preferential excitation of the Q_3,4_, Q_2_, and Q_1_ excited-state manifolds, respectively. Regions of pump scatter have been omitted from the plots.

Fig. 4 shows the transient absorption (TA) spectra of Mn(acac)_3_ in ethanol following photoexcitation at 460 nm, 640 nm, and 850 nm, which provide preferential access to the Q_3,4_, Q_2_, and Q_1_ excited states, respectively (TA spectra for all other excitation wavelengths used in this study are provided in Section S4 of the SI). Despite the differing electronic origins, excitation at all three wavelengths leads to the formation of a long-lived transient feature (in varying intensities and discussed further in Section 3.3.4.) centred near 430 nm within the first tens of picoseconds. In all cases, this band initially appears red-shifted relative to its final position, with the magnitude of the shift increasing with excitation energy (*λ*^460nm^ ≈ +5 nm, *λ*^640nm^ ≈ +4 nm, *λ*^850nm^ ≈ +2 nm). Across the excitation range 380–940 nm, the largest shift (∼ +12 nm) is observed for 400 nm excitation, while a +2 nm shift is present at 940 nm (see Section S5, Fig. S14 of the SI). This behaviour is consistent with a vibrational relaxation process, undergoing redistribution of excess energy and cooling towards the minimum geometry. Despite small differences in the magnitude of the shift, the spectra immediately after the instrument response function (IRF) are similar in shape, indicating that internal conversion from higher-lying states is complete within the instrument response and rapidly leads to population of the lowest (Q_1_) excited state.

#### 3.3.2. Relaxation kinetics

**Fig. 5:**
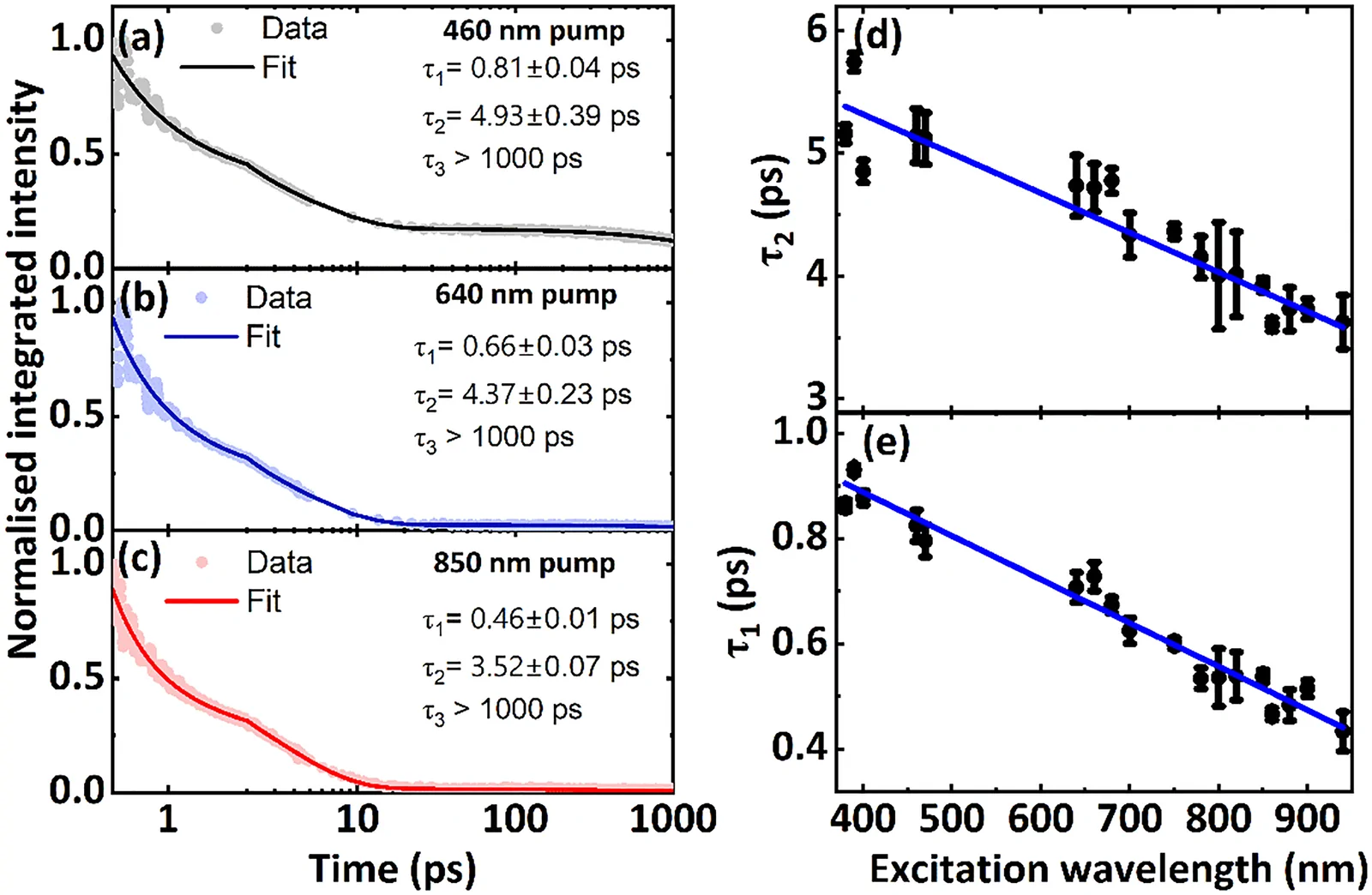
Excitation-wavelength-dependent kinetics and coherent vibrational dynamics of Mn(acac)_3_. (a)–(c) Normalised TA kinetics integrated over a probe region of 420–450 nm following excitation at 460, 640, and 850 nm, with fits shown as solid lines and extracted time constants indicated. (d) and (e) The fitted time constants are plotted against the excitation wavelength, highlighting a linear decrease in both the *τ*_2_ (panel d) and *τ*_1_ (panel e) components as excitation wavelength increases. Solid blue lines represent linear fits to the data.

The transient absorption kinetics were compared across the same range of pump wavelengths. As depicted in Fig. 5(a–c), the ultrafast relaxation kinetics following excitation to the three manifolds (Q_1_, Q_2_ and Q_3,4_) exhibit an excitation-wavelength dependence in τ_1_ and τ_2_, becoming progressively faster with increasing excitation wavelength. While the extracted time constants are similar across the investigated excitation wavelength range, both τ_1_ and τ_2_ exhibit a systematic variation with excitation wavelength. The observed trend is reproducible across the complete dataset and exceeds the associated fitting uncertainties, supporting a subtle excitation-wavelength dependence of the relaxation dynamics.

For all wavelengths, a common *τ*_3_ ≥ 1 ns is observed, which is assigned to the compressed state lifetime (further discussed in Section 3.3.4). For 460 nm excitation, the fitted time constants are *τ*_1_ = 0.81 ± 0.04 ps and *τ*_2_ = 4.93 ± 0.39 ps. At 640 nm, these decrease to *τ*_1_ = 0.66 ± 0.03 ps and *τ*_2_ = 4.37 ± 0.23 ps, while direct excitation into Q_1_ at 850 nm gives *τ*_1_ = 0.46 ± 0.01 ps and *τ*_2_ = 3.52 ± 0.07 ps. The extracted time constants for the full experimental range (380–940 nm) are plotted as a function of pump wavelength in Fig. 5(d) and 5(e). Both *τ*_1_ and *τ*_2_ exhibit a linear decrease as the excitation wavelength increases. Because the electronic relaxation to the lowest-lying excited surface is entirely complete within the IRF (as explained in Section 3.3.1) and because the extracted time constants exhibit a smooth and continuous dependence on excitation wavelength, the observed trend (*τ*^460nm^ > *τ*^640nm^ > *τ*^850nm^) in relaxation times likely reflects a vibrational phenomenon on the Q_1_ potential energy surface, rather than an electronic cascade from higher-lying states. In this framework, sub-IRF internal conversion places the system onto different regions of the same lower-lying JT-active manifold, depending on the initial excitation wavelength. At higher excitation energies, vibrational relaxation proceeds through a broader manifold of accessible configurations, potentially including regions of the surface with weaker vibronic coupling or transient trapping in shallow distortions. These effects slow the redistribution and dissipation of excess energy, delaying relaxation along the JT coordinate and, consequently, access to the conical intersection that mediates electronic relaxation to the ground state. By contrast, excitation into lower-lying states prepares the wavepacket closer to the minimum-energy geometry, reducing the extent of nuclear relaxation required before reaching the conical intersection and facilitating more rapid ground-state recovery. As we do not see ground-state bleach contributions, the majority of the vibrationally relaxed trajectories that approach the conical intersection return to the ground elongated state configuration.

We note that wavelength-dependent relaxation dynamics can, in some systems, arise from inhomogeneous broadening or ground-state aggregation, particularly at the red edge of an absorption band.^33–35^ However, this interpretation appears inconsistent with the present data, where the transient dynamics evolve smoothly across multiple absorption bands and excitation wavelengths rather than being confined to a localised red-edge region. Instead, the observed spectral evolution is more readily explained by selective excitation of different regions of the excited-state potential energy surface. This assignment is further supported by concentration-dependent UV-Vis measurements (see S2 of the SI), which reveal linear scaling of the entire absorption spectrum with concentration and no evidence of aggregation up to 50 mM. While we cannot completely exclude contributions from aggregation or other forms of static heterogeneity, the available evidence does not indicate that these effects dominate the observed wavelength-dependent dynamics.

#### 3.3.3. Coherent dynamics

**Fig. 6:**
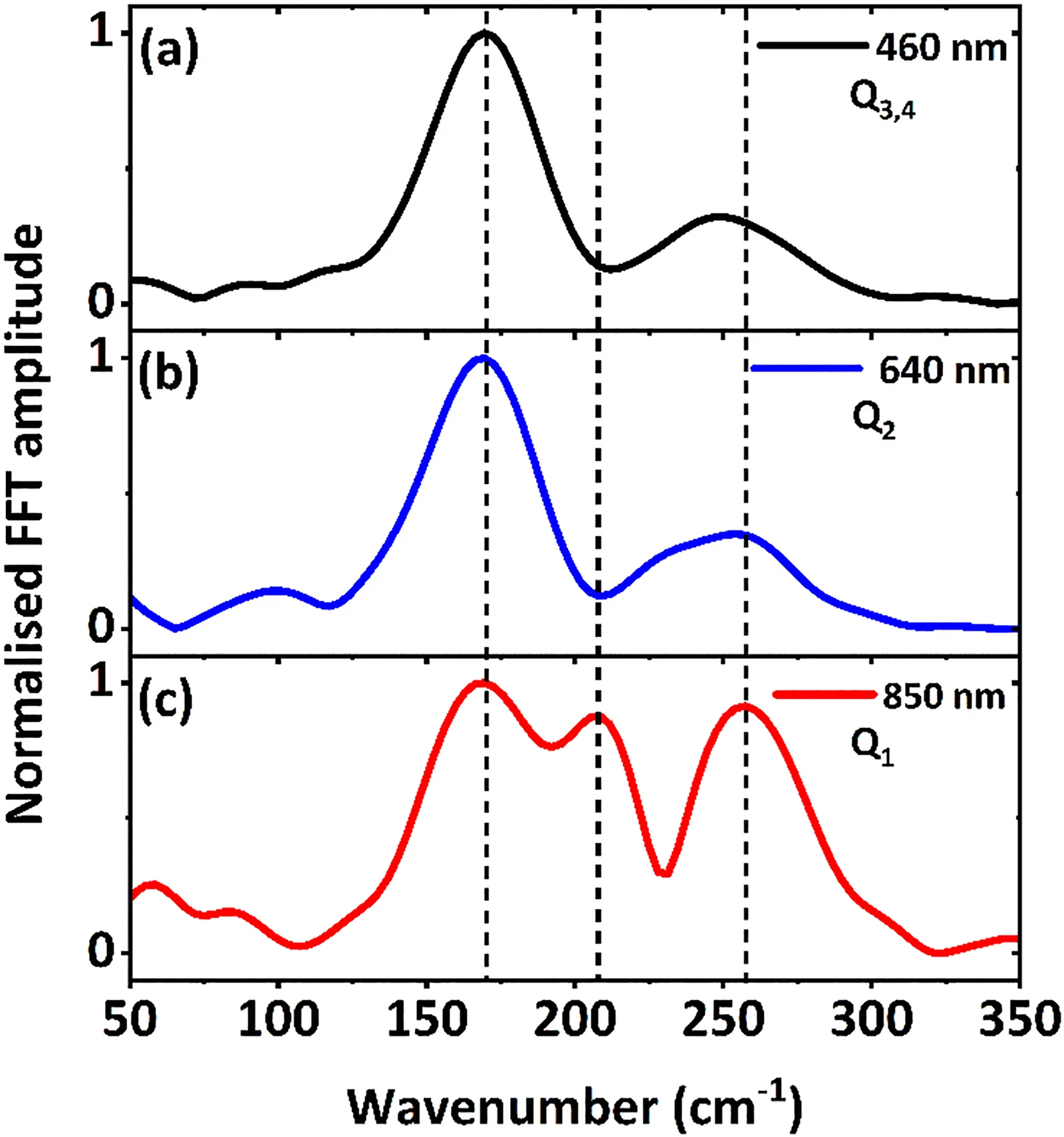
FFT amplitudes of the residual oscillations after subtraction of the population dynamics, following excitation to the (a) Q_3,4_ excited state at 460 nm, (b) Q_2_ excited state at 640 nm, and (c) Q_1_ excited state at 850 nm. Dashed lines mark the dominant frequencies at 170, 208, and 254 cm^−1^. The data from the 0.3–2.0 ps temporal window were Hann-windowed and zero-padded prior to performing FFT with a 10 fs step size, resulting in a theoretical spectral resolution of 19.6 cm^−1^.

Oscillations superimposed on the kinetic traces (Fig. 5a–c) were analysed by performing FFT analysis on the residuals of the kinetic fits. As shown in Fig. 6a–c, excitation at 460 and 640 nm yields oscillations at two main frequencies at 170 and 254 cm^−1^, while excitation at 850 nm reveals an additional mode at 208 cm^−1^. The selective appearance of the 208 cm^−1^ mode upon Q_1_ excitation suggests that it is a vibrational signature of the Q_1_ excited state and is assigned to a JT-active normal mode on the basis of vibrational frequency calculations (see Section S3.4 of the SI). These observations are consistent with previous reports on Mn(dpm)_3_ following 940 nm photoexcitation,^16^ where three frequencies at 110, 155, and 180 cm^−1^ were assigned to the ground elongated, Q_1_ excited, and ground compressed JT-active vibrations, respectively. By analogy, the 170 cm^−1^ mode observed here is consistent with an elongated ground state JT-vibration, while the higher-frequency 254 cm^−1^ mode is assigned to motion associated with the compressed configuration. The spectral evolution and FFT analyses together show that while different excitations access the same compressed state within the IRF, the transformation likely occurs *via* alternative pathways. Notably, the persistence of coherent vibrational motion during the formation of the compressed state indicates that the underlying structural transformation occurs on a timescale comparable to, or faster than, the vibrational period. Previous work on Mn(dpm)_3_ shows the compressed state is formed within ¼ of the JT vibrational period, and for Mn(acac)_3_ this would correspond to ∼32 fs, which is within our IRF. This ultrafast timescale implies that the system reaches the crossing region between Q_0_ and Q_1_ ballistically, prior to significant intramolecular vibrational energy redistribution (IVR).

#### 3.3.4. Compressed-state yields

To assess changes in the population that converts to the compressed state following passage of the CI, we define a compressed state ratio for the JT switch as shown in eqn (2). This definition is used because the extinction coefficients of both the excited and compressed states are unknown. Since the measured change in optical density depends on both the species population, extinction coefficient, and the experimental conditions (*i.e.* pump fluence, pump-probe overlap, sample concentration), the absence of these values prevents determination of a quantum yield. However, wavelength-dependent changes in this ratio directly report on changes in the population of the compressed state.2
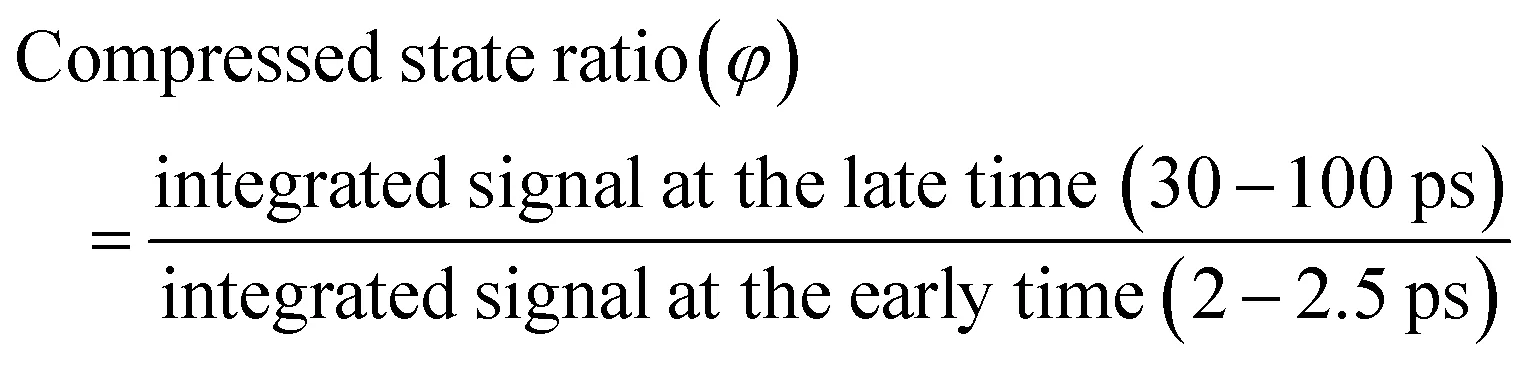


**Fig. 7:**
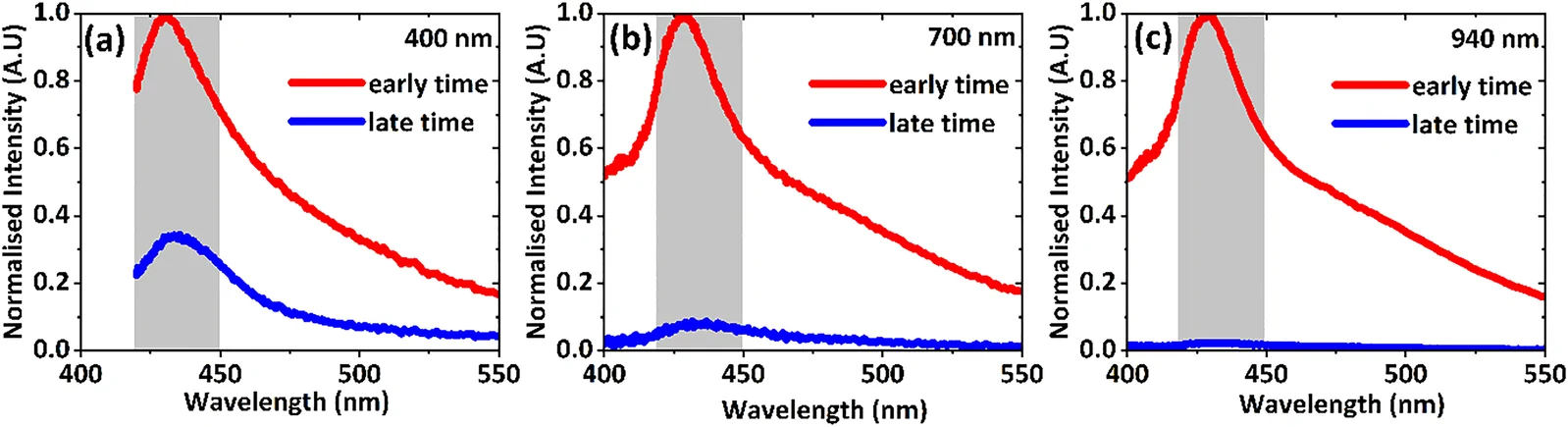
Early- and late-time TA spectra of 50 mM Mn(acac)_3_ in ethanol following excitation at (a) 400, (b) 700, and (c) 940 nm. Early-time spectra are averaged over 2–2.5 ps, and late-time spectra over 30–100 ps. The grey region marks the 420–450 nm integration window used.

**Fig. 8:**
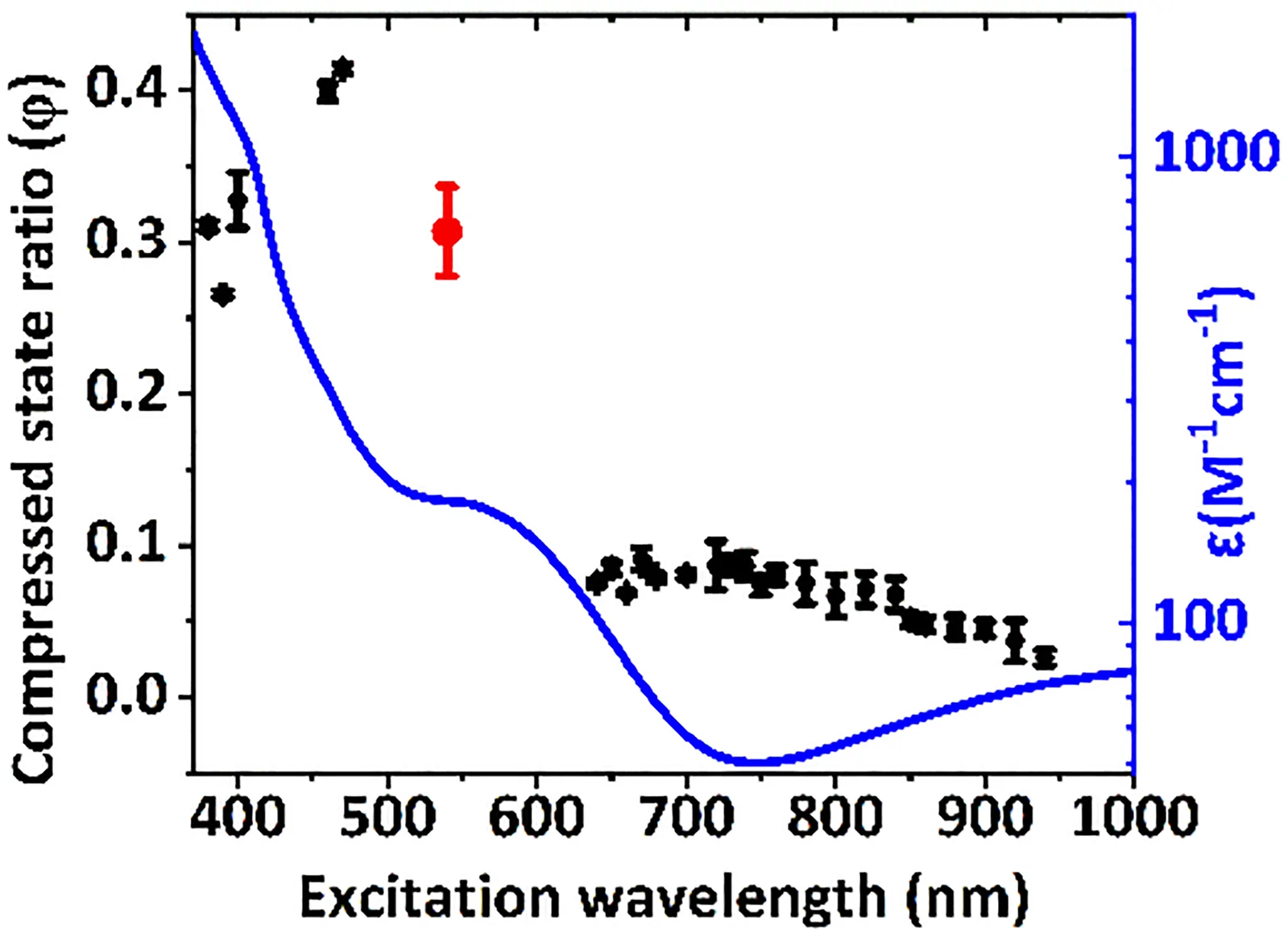
Excitation energy dependence of the ratio of compressed photoproduct to the excited state absorption band for photoexcitation in 380–940 nm region. Black dots show experimental data points in this study. The red dot shows data extracted from Liedy *et al.*^13^ The error bars represent the standard deviation across multiple measurements, with uncertainties propagated from both the early-time and late-time averaging. Solid blue line shows the ground state absorption spectrum of Mn(acac)_3_ reproduced from Fig. 2(a).

Eqn (2) is expressed as the ratio of the integrated transient absorption signal at late times (30–100 ps), where the signal has plateaued (Fig. 5a) and reflects the compressed-state population, to that at early times (2–2.5 ps), which captures the initially prepared excited-state population for a period after IVR but prior to any significant electronic relaxation. Although the wavepacket may reach the CI within ∼32 fs (a quarter of the vibrational period), this early transfer remains small relative to the total excited-state population across most of the excitation range, so the early-time signal serves as an approximately constant normalisation reference. Averaging over these time windows improves signal-to-noise and avoids contribution from coherent oscillations. We confirm the validity of these windows by comparing with different time windows, and we find very little difference in trend (Fig. S15 of the SI). These comparisons span early-time windows capturing varying extents of band shifting and IVR; in each case, the resulting differences appear as a uniform offset in signal amplitude, consistent with ongoing population decay, rather than a change in spectral shape, and since our interpretation relies on the wavelength-dependent trend rather than absolute ratio values, this offset does not affect the conclusions drawn. Representative early- and late-time spectra for three excitation wavelengths are shown in Fig. 7. The ratio is determined by integrating the signal over the 420–450 nm region (highlighted in grey in Fig. 7 and remains free of pump scatter). The resulting ratios, plotted as a function of excitation wavelength, are shown in Fig. 8. As some of the early-time integrated intensity has already decayed to form the compressed state, a slight error is expected in the ratios at higher excitation energies (380–470 nm). However, these ratios remain substantially larger than those observed for excitation in the 640–940 nm range, such that this systematic bias does not significantly affect the overall trend.

Fig. 8 shows that the ratio of the compressed state depends strongly on excitation wavelength. In the lower excitation energy region (940–640 nm), the ratio exhibits a gradual monotonic increase with increasing excitation energy from 940–700 nm. This initial rise is followed by a distinct plateau between 700–640 nm. Because the intermediate excitation (640–470 nm) region was experimentally inaccessible, data extracted from Liedy *et al.*^13^ at 540 nm are included; this point bridges the values observed at 640 nm and 470 nm, confirming another upward trend with increasing excitation energy. The ratio reaches a pronounced global maximum at 470 nm. At higher excitation energies (460–380 nm), the ratio decreases and fluctuates, though it remains much higher compared to the 940–640 nm region.

To understand this wavelength dependence, we first consider excitation into the Q_1_ excited state (700–940 nm). Because the compressed geometry is accessed *via* nuclear motion along the JT-active coordinate on the excited-state surface, increasing excitation energy leads to greater excess vibrational energy deposited into the system. This total excess energy is calculated as, *E*_excess_ = *E*_excitation_ − *E*_CI_. Where, *E*_excitation_ is the excitation energy and *E*_CI_ is the energy of the conical intersection, obtained from quantum chemical calculations (see Fig. 2b). Following this initial vertical excitation, *E*_excess_ rapidly distributes across the vibrational manifold, with a defined fraction, (*A*), populating the reactive JT coordinate. Thus, the excess energy available along this reaction coordinate is *E*_rc_ = *AE*_excess_. The value of *A* depends on the strength of vibronic coupling described by the Huang–Rhys (HR) factor,^36^ which determines how strongly the electronic excitation projects onto that normal mode and can be obtained from quantum chemical calculations.^37–39^ For direct photoexcitation into the Q_1_ state of Mn(acac)_3_, the HR factor for the JT-active mode is 1.120, corresponding to *A* = 0.1116, or 11.16% of the excess being deposited into this coordinate. This was obtained from our mode-resolved decomposition of the excited-state reorganisation energy calculations, as discussed in SI Section S3.5. The results from these calculations are shown in Table S5 of the SI. Within the 1D LZ model (eqn (1)), the compressed state ratio, and hence the yield, depends on the excess energy deposited into the reactive JT coordinate (*E*_rc_) as shown in eqn (3). Where, *B* is a scaling factor and *C* consolidates the constant theoretical parameters. Further derivation of eqn (3) is described in ref. [12] and outlined in Section S7 of our SI.3
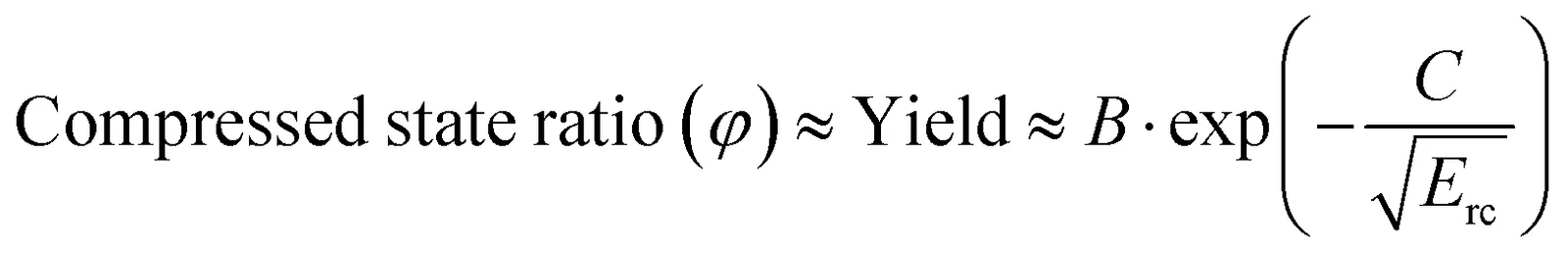


**Fig. 9:**
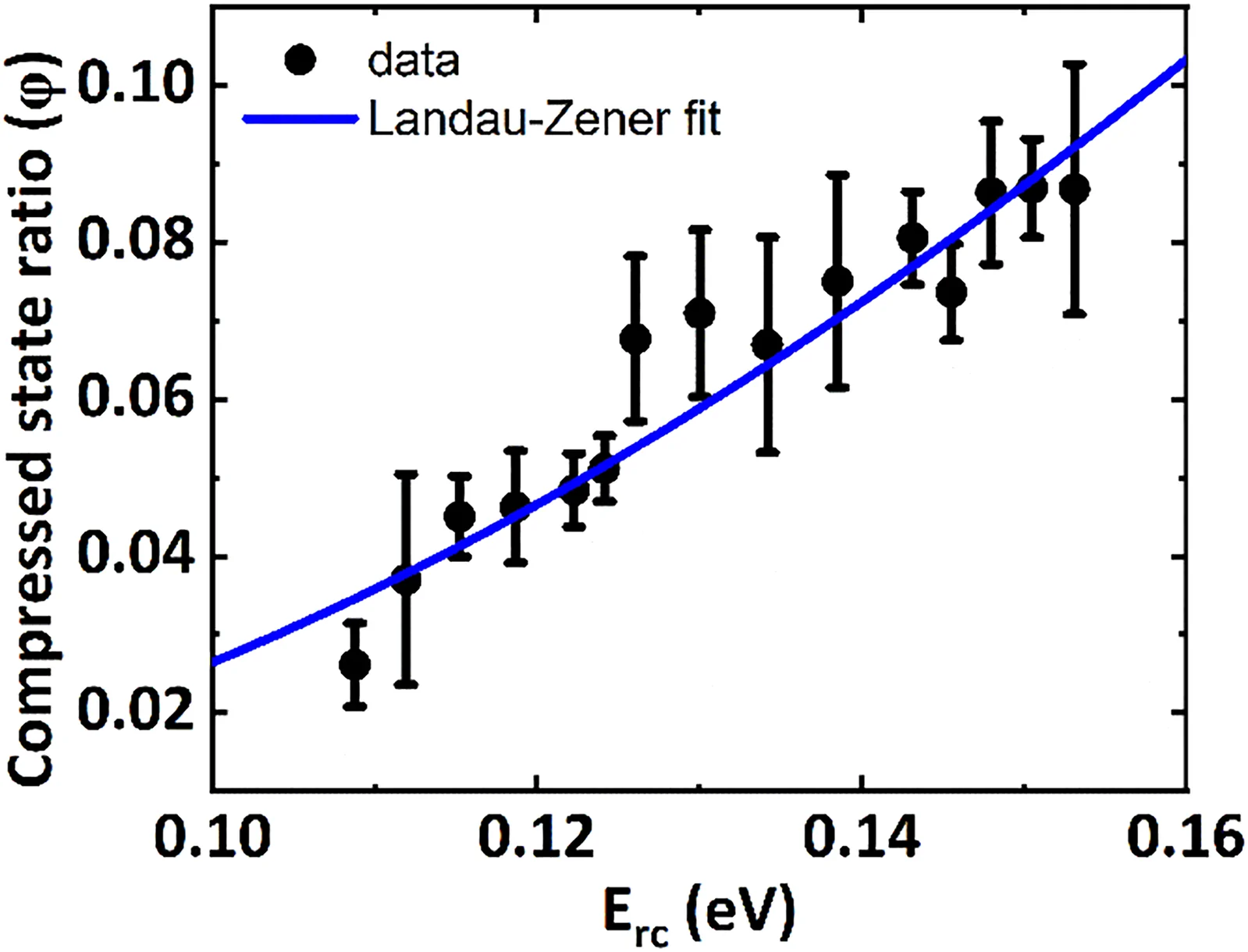
Compressed-state ratio, (*φ*) as a function of excess energy deposited into the reactive JT coordinate, *E*_rc_, for excitation in the Q_1_ region. Black circles show the experimental data, and the blue line shows the Landau–Zener fit using eqn (3).

Fig. 9 shows the compressed state ratio as a function of excess energy deposited into the reactive JT coordinate, *E*_rc_, for the 940–700 nm region and is fitted to eqn (3). The excellent agreement to the fit suggests the yield is governed by the velocity-dependent probability at a single nonadiabatic crossing, driven by 1D motion along the JT coordinate. The plateau observed between 640 and 700 nm (Fig. 8) coincides with the onset of Q_2_ absorption, where the system begins to access a different excited state. As the relaxation pathway from Q_2_ to the compressed state differs from that of direct Q_1_ excitation, such that it involves an additional internal conversion step prior to motion along the JT coordinate, the simple relationship between initial photon energy and wavepacket velocity at the crossing region breaks down. Consequently, it is more likely that excess energy partitions into non-reactive modes and the yield no longer increases monotonically, producing the observed plateau. A similar observation was observed in the *cis*–*trans* isomerism of rhodopsin.^11^ Excitation at higher photon energies (540–380 nm) produces a sharper increase in yield, signalling a breakdown of this one-dimensional model. This enhancement at elevated energies suggests the activation of additional reactive pathways, potentially involving higher-lying electronic states or multi-dimensional nuclear trajectories that more efficiently funnel the wavepacket through the conical intersection towards Q_0_. This interpretation is supported by the disappearance of the 208 cm^−1^ vibrational mode previously assigned to the JT-reactive coordinate under high-energy excitation (see Fig. 6c). Its absence suggests that the dynamics no longer proceed predominantly along this mode but instead involve alternative nuclear motions. The yield reaches a maximum near 470 nm and decreases at even higher excitation energies. This non-monotonic behaviour deviates from simple LZ expectations and suggests a multidimensional nature to the excited-state dynamics. The resulting increase and decrease in yield therefore reflect a more complex interplay between excitation energy, nuclear motion, and nonadiabatic coupling on the excited-state landscape.

## 4. Conclusion

This work has examined the excitation-energy dependence of the ultrafast JT switching dynamics in Mn(acac)_3_ using transient absorption spectroscopy. By selectively preparing the Q_1_, Q_2_, and Q_3,4_ excited-states, we demonstrate that formation of the compressed-state photoproduct occurs *via* pathways that depend strongly on the initially accessed electronic state, despite converging to a common final compressed state structure. Analysis of the transient spectra, relaxation kinetics, and coherent vibrational dynamics shows that excitation into Q_1_ primarily drives motion along the JT active coordinate, whereas excitation into Q_2_ and Q_3,4_ introduces additional relaxation steps and access to a broader range of nuclear configurations, leading to slowing of dynamics.

Following excitation into Q_1_, the increase in the compressed-state yield with increasing excitation energy is consistent with a velocity-dependent nonadiabatic transition described by a one-dimensional LZ framework. Excitation into Q_2_ leads to a plateau in the yield, indicating that the relationship between photon energy and wavepacket velocity along the JT coordinate is disrupted by intermediate relaxation processes. In contrast, excitation into the higher-lying Q_3,4_ manifold results in a markedly larger compressed-state yield, accompanied by non-monotonic behaviour as a function of excitation energy. This substantial increase in yield indicates that access to these higher-lying states enables more efficient population transfer to the compressed-state minimum, likely through multidimensional nuclear dynamics and additional relaxation pathways that more effectively funnel population to the compressed state.

The combined experimental results support a picture in which the outcome of the JT switch is governed not only by the topology of the potential energy surfaces, but also by the initially accessed electronic state and the resulting nuclear wavepacket dynamics. While a one-dimensional LZ description captures the behaviour following excitation into Q_1_, the dynamics following excitation into Q_2_ and particularly Q_3,4_ require consideration of multidimensional effects and mode-specific energy redistribution. More broadly, these results highlight excitation energy as an effective experimental parameter for influencing nonadiabatic structural dynamics in transition metal complexes. This provides insight into how ultrafast structural transformations may be steered through selective population of electronic states, with potential implications for the design of photoresponsive molecular systems where control over structural and magnetic properties is desirable.

## Author contributions

B. P. performed the experiments, conducted the analysis, interpreted the data, and drafted the manuscript. J. E. performed the quantum chemical calculations. K. B. contributed to data interpretation. A. G. supported B. P. with data analysis and manuscript preparation. O. J. and T. P. acquired funding and contributed to conceptualisation. R. P. supported B. P., conceptualised and managed the project, interpreted the data, drafted and edited the manuscript. All authors reviewed and approved the final manuscript.

## Conflicts of interest

There are no conflicts to declare.

## Supplementary Material

CP-028-D6CP02713J-s001

## Data Availability

The datasets supporting this paper are openly available from eData, the STFC Research Data repository at https://doi.org/10.5286/edata/981. Supplementary information (SI) is available. See DOI: https://doi.org/10.1039/d6cp02713j.
