# Supplementary material for: Excitation energy dependence of ultrafast Jahn–Teller switching in Mn(iii) acetylacetonate
Source: Phys Chem Chem Phys. 2026 Aug 26;28(36):22003–12. doi: 10.1039/d6cp02713j (PMC13599768; doi:10.1039/d6cp02713j)
Supplement: Supplementary file 1 [file CP-028-D6CP02713J-s001.pdf]

Supplementary Information (SI) for Physical Chemistry Chemical Physics.
This journal is © the Owner Societies 2026

Supplementary information

Excitation Energy Dependence of Ultrafast Jahn–Teller Switching in Mn(III) Acetylacetonate

Binoy Paine,[1] Julien Eng,[2] Kyle Barlow,[1] Thomas J. Penfold,[2] J. Olof Johansson,[1] Alice E. Green,[1] Ryan Phelps*[3]

[1] EaStCHEM School of Chemistry, University of Edinburgh, David Brewster Road, EH9 3FJ, Edinburgh, UK
[2] Chemistry, School of Natural and Environmental Sciences, Newcastle University, Newcastle upon Tyne, UK.
[3] STFC Central Laser Facility, Research Complex at Harwell, Rutherford Appleton Laboratory, Harwell Campus, Didcot, OX11 0QX, UK. Email: ryan.phelps@stfc.ac.uk

Table of Contents

S1 UV-Vis measurements and Transient absorption spectroscopy (TAS) of Mn(acac)3 2

S2 Pump spectra and Fluence dependence measurements 4

S3 Quantum chemical calculation 5

S3.1 Computational details 5

S3.2 Electronic structure calculation 7

S3.3 Potential energy surface scan details 8

S3.4 Vibrational Frequency Calculations and Mode Assignment 9

S3.5 Huang–Rhys (HR) factors and mode-resolved reorganisation energy 9

S4. Full Transient Absorption Dataset 13

S5. Spectral Shift of the TAS Feature 15

S6. Time-Window Sensitivity of the Relative compressed state yield 16

S7. Landau–Zener Model and Derivation of Equation 3 16

S1 UV-Vis measurements and Transient absorption spectroscopy (TAS) of Mn(acac)3

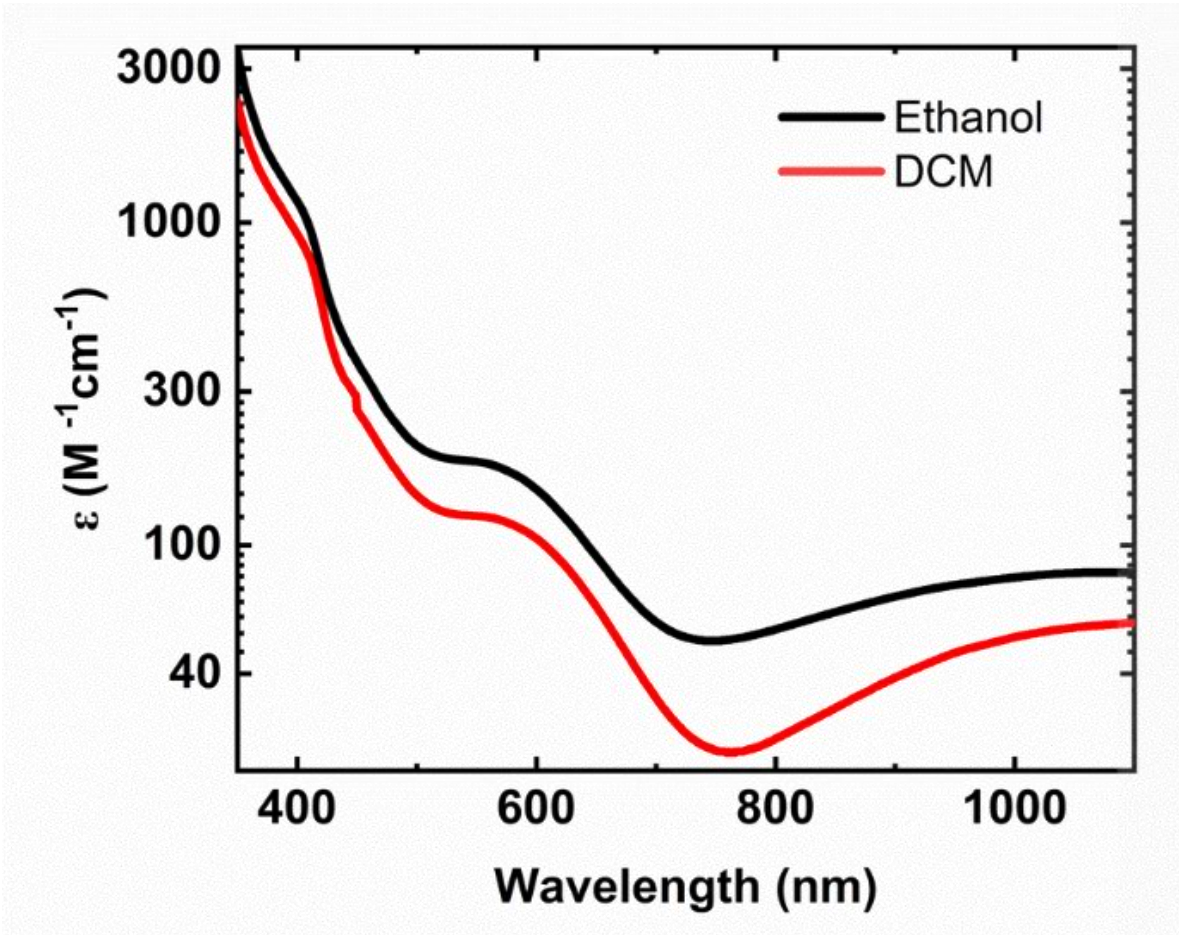

Figure S1: UV-Visible absorption spectrum of Mn(acac)$_3$ in ethanol (black solid lines) and dichloromethane (red solid line) at room temperature recorded with Shimadzu UV-1800 spectrophotometer.

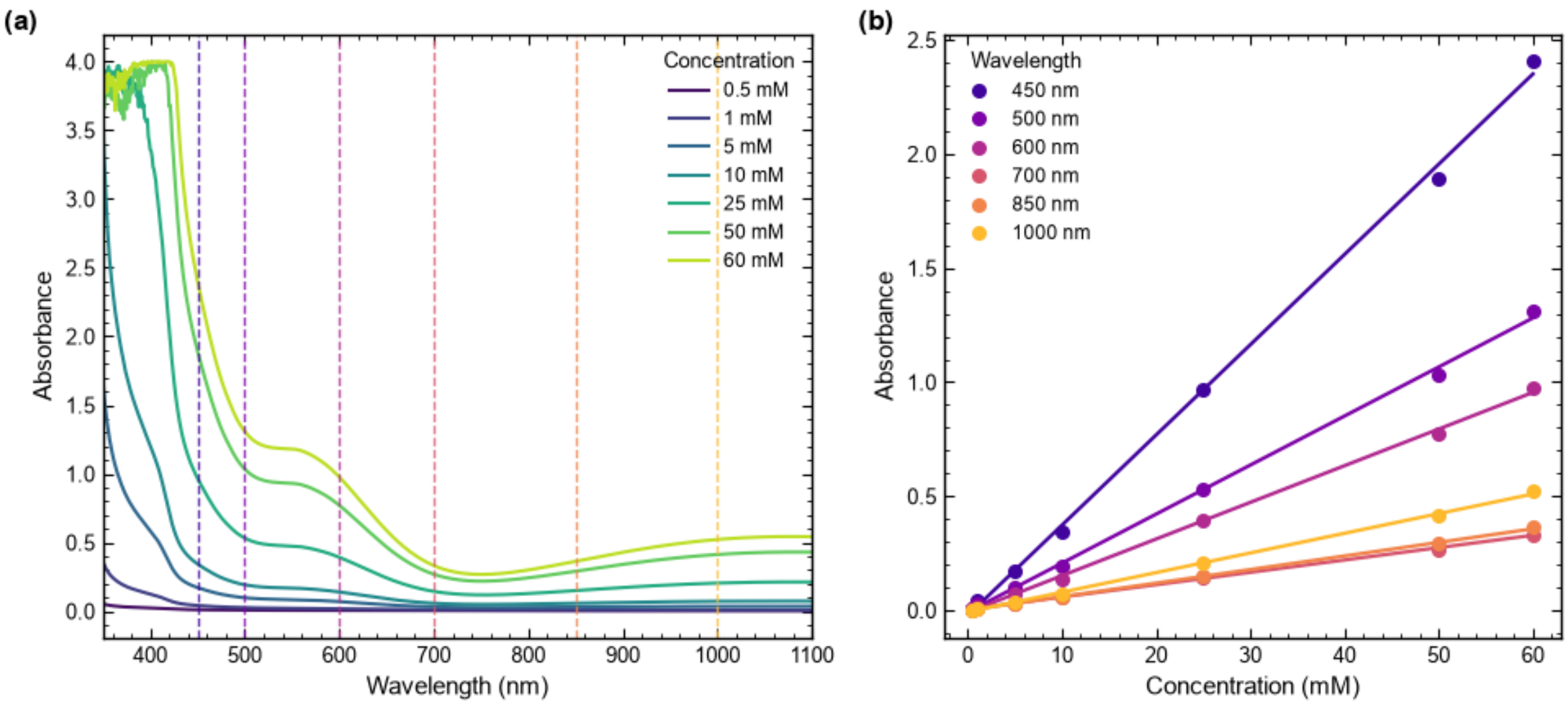

Figure S2: (a) Concentration-dependent UV–visible absorption spectra of Mn(acac)$_3$ in ethanol over the concentration range 0.5–60 mM, recorded at room temperature using a 1 mm quartz cuvette and a Shimadzu UV-1800 spectrophotometer. (b) Absorbance at selected wavelengths plotted as a function of concentration and fitted using linear regression. We acknowledge that the absorbance exceeds 1 OD, a regime in which non-linear effects can occur. However, the manufacturer specifies that the spectrometer response remains linear up to at least 2 OD.

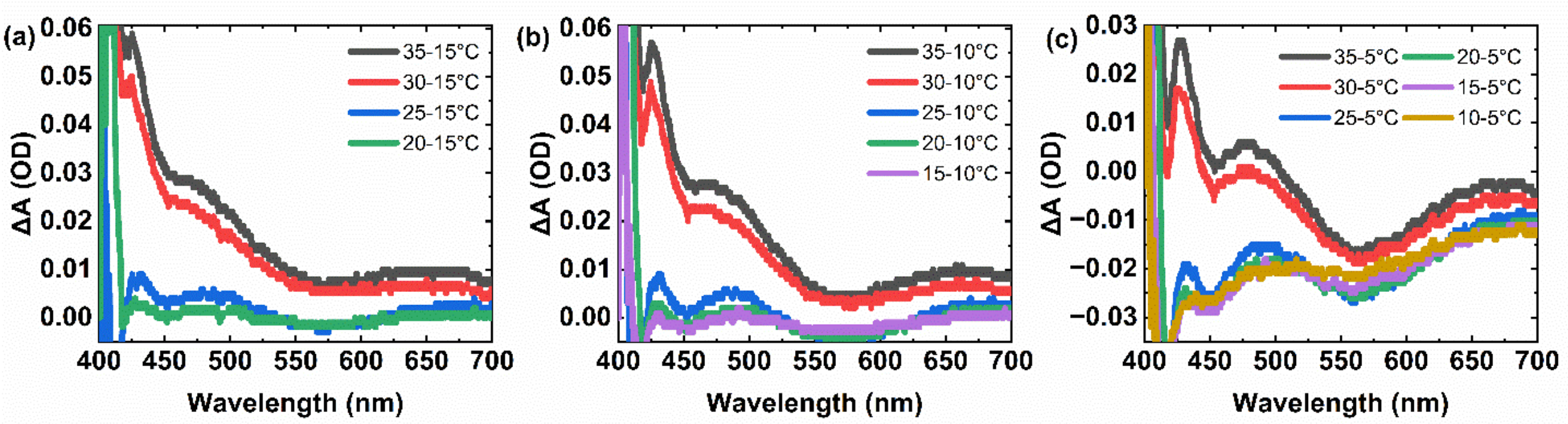

Figure S3: Temperature-dependent UV-Vis difference spectra recorded using a Shimadzu UV-1800 spectrophotometer, calculated relative to reference spectra recorded at (a) 15 °C, (b) 10 °C, and (c) 5 °C. Measurements below 15 °C are increasingly affected by condensation on the cuvette and optical surfaces, leading to baseline distortions. At temperatures above 35 °C, solvent evaporation and boiling become significant, likewise causing baseline instability. These artefacts limit the experimentally accessible temperature range.

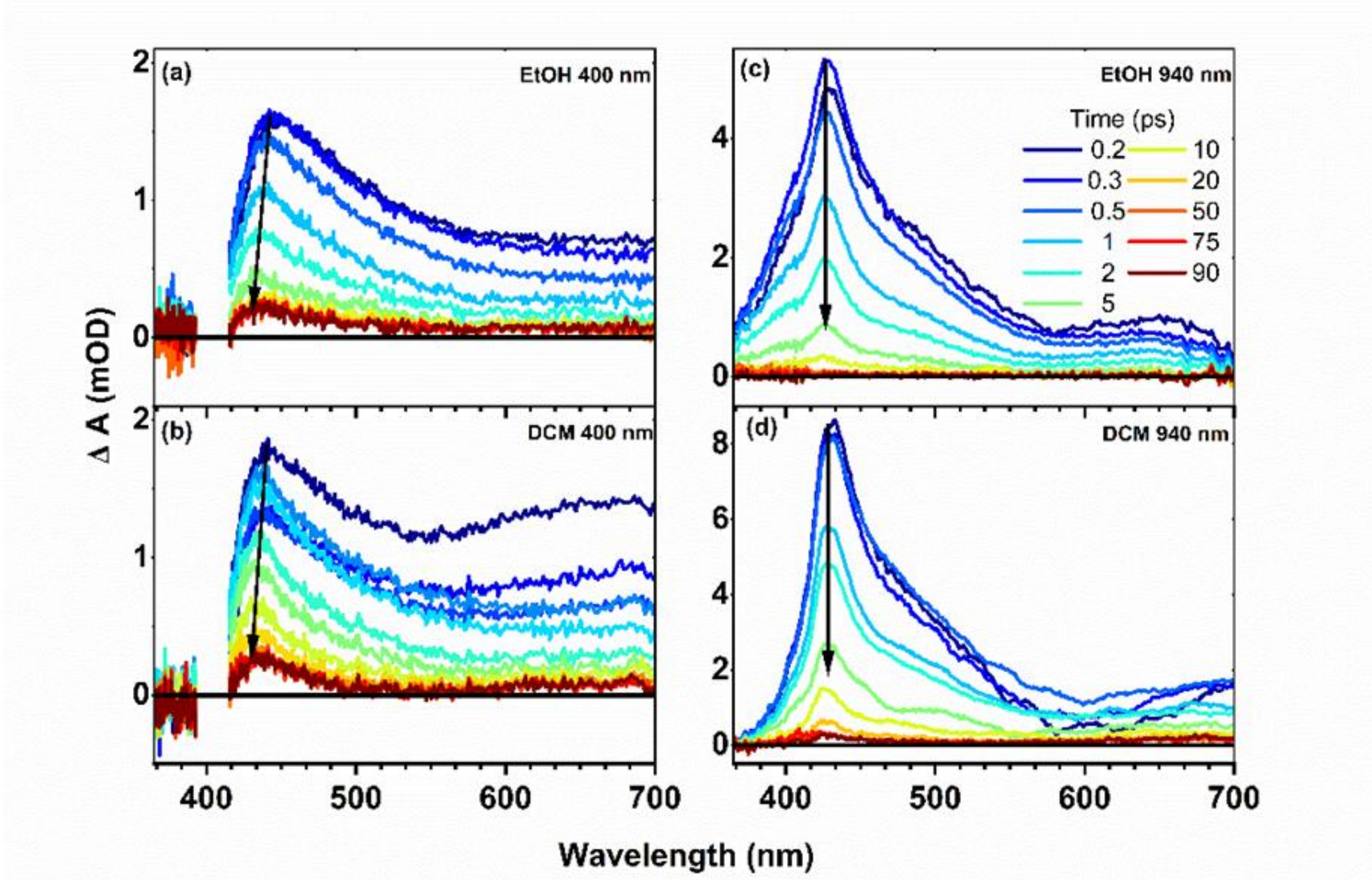

Figure S4: Transient absorption difference spectra of $Mn(acac)_3$ following photoexcitation at 400 nm in (a) ethanol and (b) dichloromethane, and at 940 nm in (c) ethanol and (d) dichloromethane

Figure S4 compares the transient absorption spectra of $Mn(acac)_3$ in ethanol and dichloromethane following excitation at 400 and 940 nm. In both solvents, excitation at either wavelength produces a transient absorption feature centred near 430 nm within the first few picoseconds. Following 400 nm excitation, a broader band initially forms at ~440 nm before evolving over ~5 ps to a narrower feature at 430 nm (Figure S4a,b), as indicated by the solid arrows. In contrast, 940 nm excitation results in the direct formation of a comparatively narrow and structured band at 430 nm, with only minimal spectral evolution (Figure S4c,d).

Overall, the spectral signatures and relaxation timescales are qualitatively similar in ethanol and dichloromethane, indicating that the primary excited-state dynamics are largely insensitive to solvent.

A notable difference, however, is observed in the amplitude of the long-lived 430 nm feature assigned to the compressed-state species. Following direct excitation of the Q_1 state at 940 nm, this feature is significantly more intense in dichloromethane than in ethanol, indicating a greater population of the compressed state in DCM. As higher-lying state relaxation is largely bypassed under these excitation conditions, the observed difference likely reflects a solvent influence on the structural evolution of the Q_1 excited-state ensemble. One possible explanation is that solvent-dependent friction affects nuclear motion along the coordinate leading to the compressed-state geometry. In the less strongly interacting DCM environment, reduced dissipation of nuclear motion may permit more efficient evolution towards the compressed-state minimum, whereas stronger solute-solvent coupling in ethanol may impede this motion and favour competing relaxation pathways. While the precise origin of this solvent dependence remains unclear, the results suggest that solvent coupling influences the branching between relaxation pathways following population of the Q_1 state, thereby affecting the yield of the long-lived compressed-state species.

S2 Pump spectra and Fluence dependence measurements

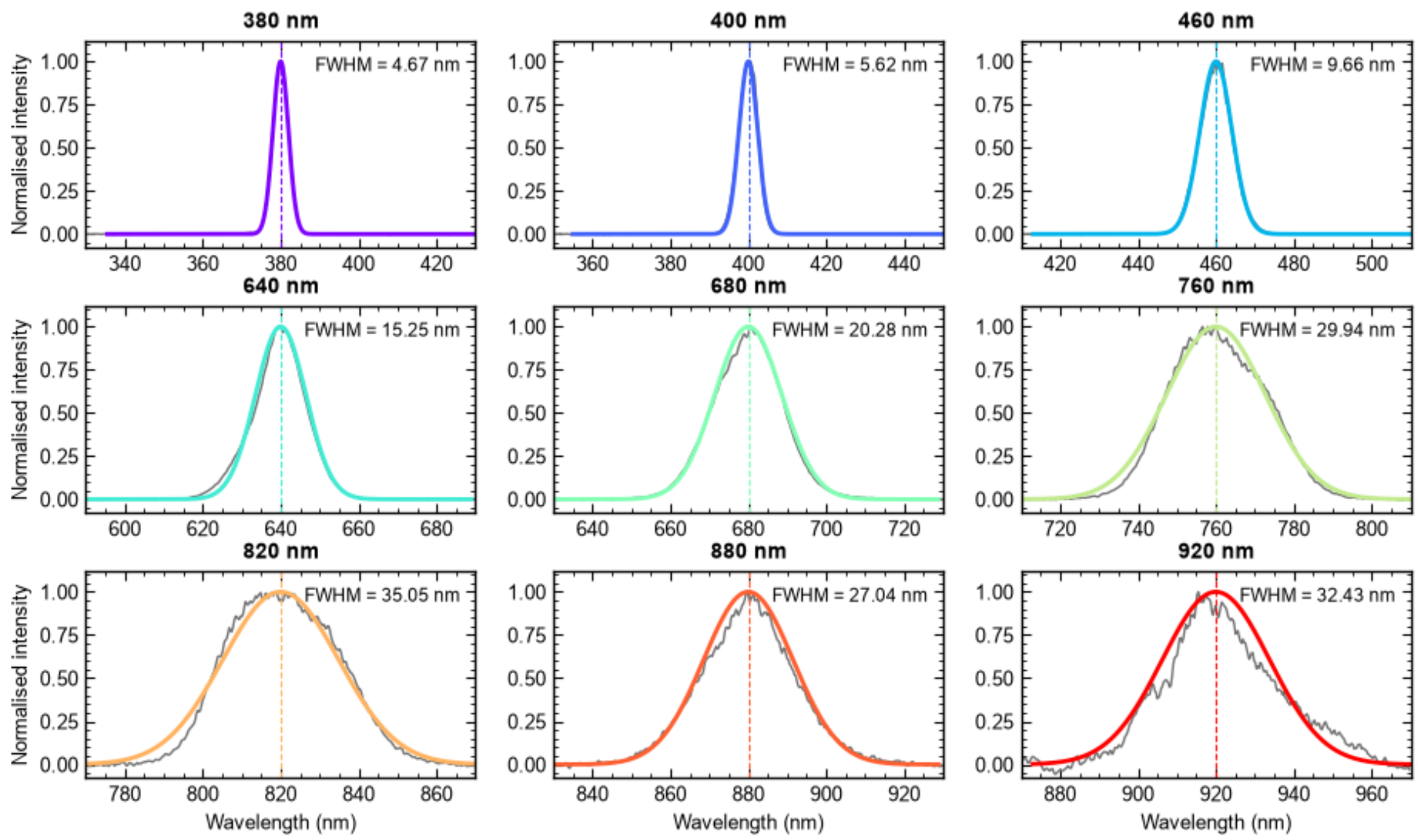

Figure S5: Pump spectra and Gaussian fits. The corresponding full width at half maximum (FWHM) values are indicated in the inset.

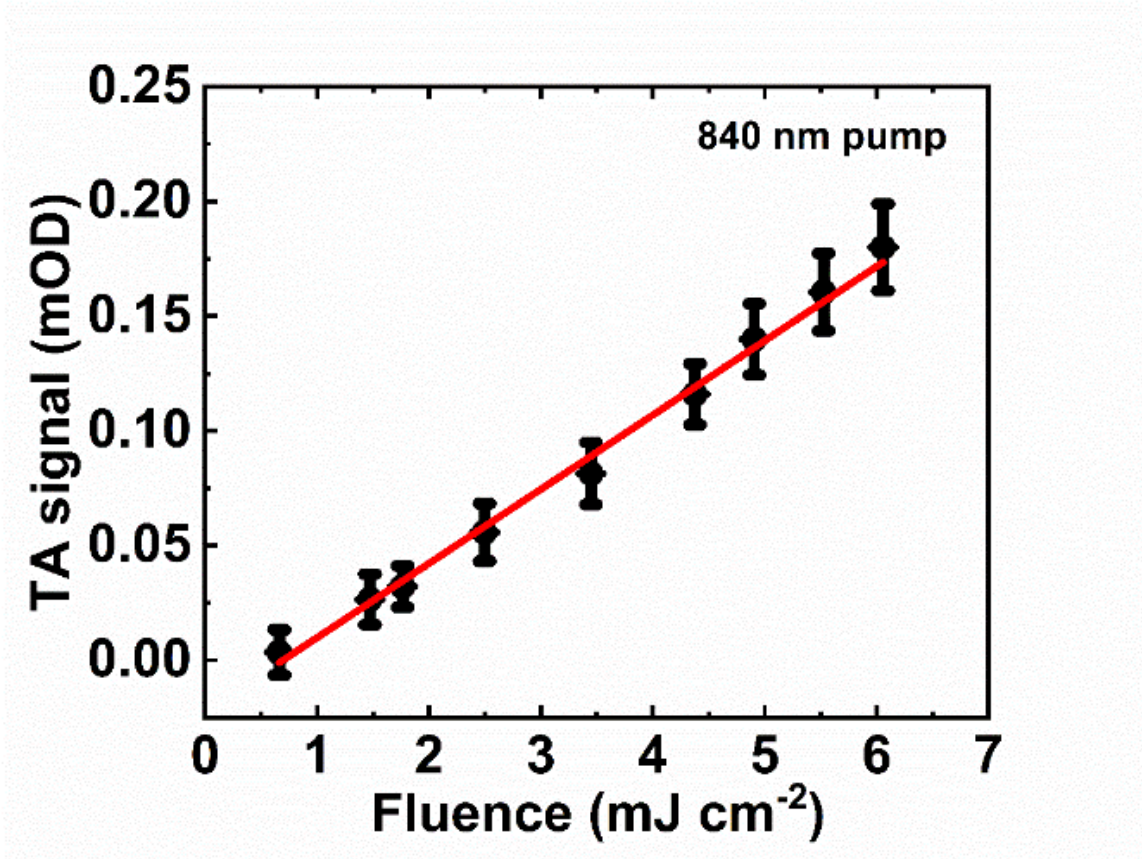

Figure S6: Fluence dependence measurements after pumping at 840 nm. The black dots and error bars show the average TA signal between 420-450 nm taken at 100 ps plotted against Fluence.

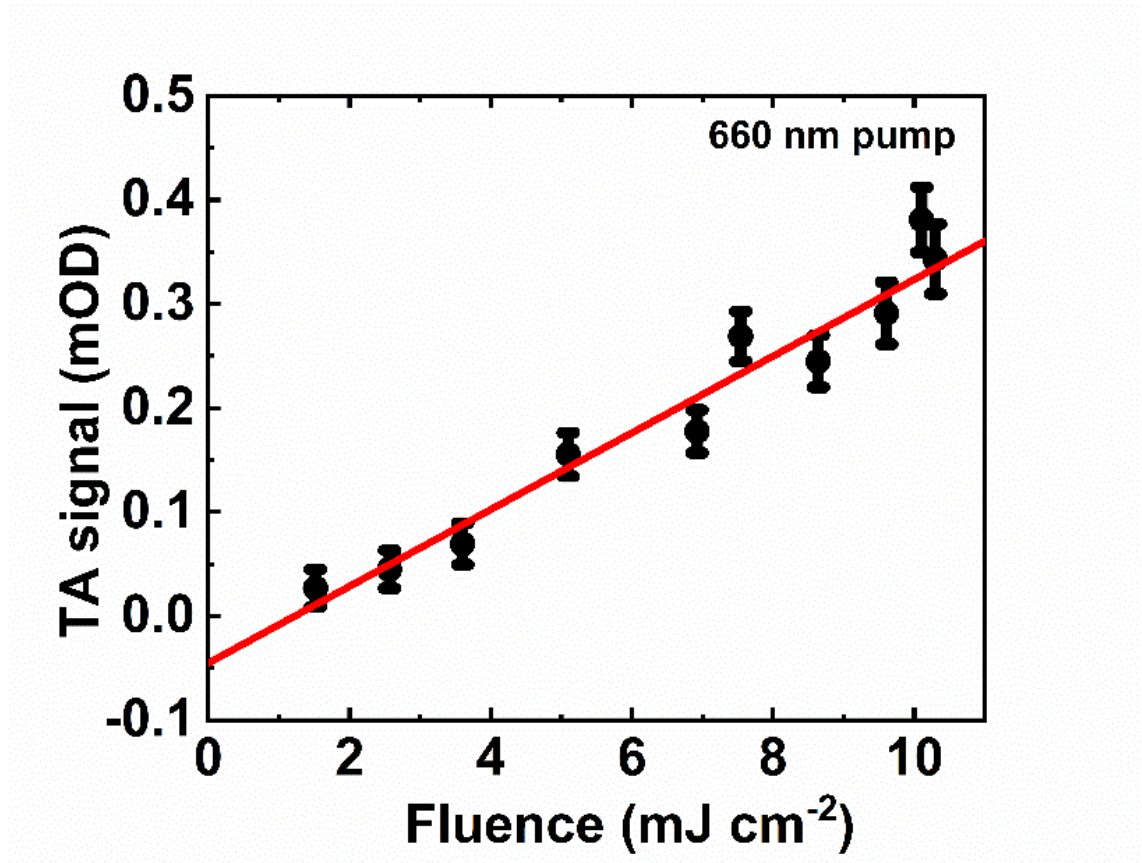

Figure S7: Fluence dependence measurements after pumping at 660 nm. The black dots and error bars show the average TA signal between 420-450 nm taken at 100 ps plotted against Fluence.

S3 Quantum chemical calculation

S3.1 Computational details

All calculations were performed using the Orca 6.1.1 quantum chemistry package [1]. Geometries were optimised using density functional theory (DFT) and the linear response time-dependent DFT (TD-DFT) in the ground and exited states, respectively. The Q_0/Q_1 conical intersection was estimated using two approaches: (1) using the gradient projection method and (2) by optimising the electronic ground state in the D_3 point group. TD-DFT is known to be unable to predict accurately conical intersections with the ground state due to its inability

to describe multi-reference character. In this case, the ground state and the first excited quintet states are of different symmetry and therefore do not mix via derivative coupling. TD-DFT is thus able to find the conical intersection between Q_0 and Q_1. The two approaches resulting in the same geometry further confirm this observation.

DFT/TD-DFT calculation were performed using the B3LYP functional[2] in conjunction with the def2-TZVP basis set[3]. Linear interpolation was performed in Cartesian coordinates between the elongated ground state minimum and the conical intersection and between the conical intersection and the compressed minimum. Electronic structure was computed at the complete active space self-consistent field method (CASSCF) with an active space composed of the four electronic in the five d orbitals of the Manganese atoms (figure S5). The state average method was employed over five quintet, 45 triplet and 50 singlet states that are all the configuration S functions possible within this active space. The N electron valence perturbation theory at the 2nd order (NEVPT2) was employed to account for some dynamic correlation.

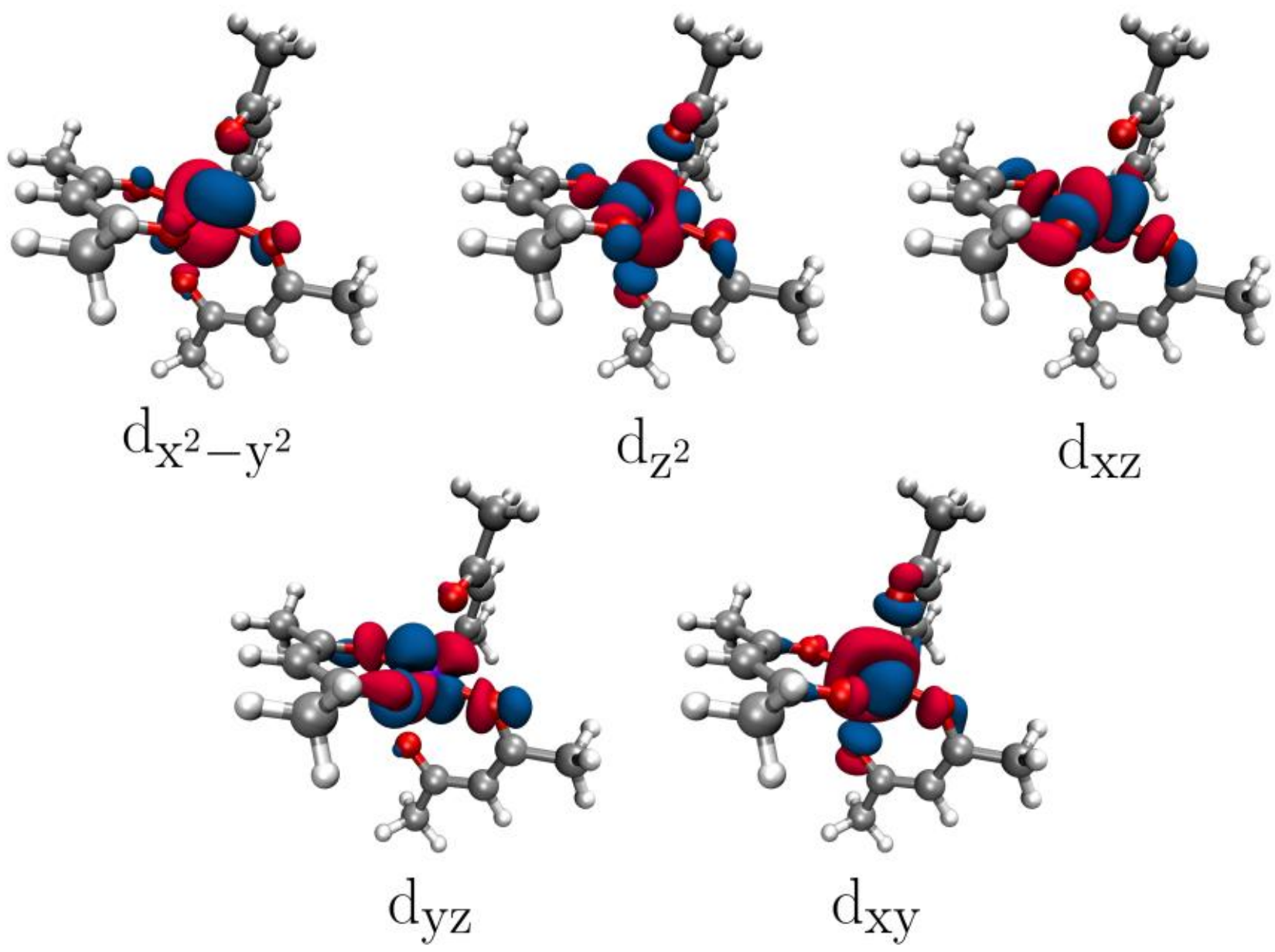

Figure S8: Molecular orbitals included in the active space for CASSCF/NEVPT2 calculations. It comprises the 4 unpaired electrons in the 5 d orbitals.

Scalar relativistic effects were included using the Douglas Kroll Hess to thee second order Hamiltonian [4-7] and the RIJCOSX approximation was used for quicker evaluation of the integrals.[5].

The irreducible representations and spin multiplicity of the electronic states included in the CAS procedures are reported in Table S1.

Ms=	5	3	1
A	3	21	28
B	2	24	22

Table S1: number of configuration state functions (CSFs) per spin multiplicity and per irreducible representations for 4 unpaired electrons in 5 d orbitals in the C_2 point group.

Huang-Rhys factor were computed in Orca using the vertical gradient approximation. The values reported in square bracket for the reorganisation energy and the Huang Rhys factors have been computed with VCMaker[9], a software developed in house, from the difference of coordinates between the conical intersection optimised geometry (q^{CI}) and the ground state optimised geometry (q^{FC}) in the dimensionless normal coordinate basis.

$$\Delta q_i = q_i^{CI} - q_i^{FC} = \frac{-\kappa_i}{\omega_i}$$

where κ is the gradient of Q_1 along normal mode q_i and ω_i is its frequency. The reorganisation energy decomposition for normal mode i then takes the form:

$$\Delta E_i = \frac{\kappa^2}{2\omega}$$

The values reported in bracket as Huang Rhys factors in Table S5 are technically not real Huang Rhys factor as they are not evaluated from the minimum of Q_1. They are therefore not directly comparable to the values derived from the gradient at Franck-Condon; however, they serve the purpose of identifying which normal modes are important to reach the seam of conical intersection and emphasising the anharmonicity along given normal modes.

S3.2 Electronic structure calculation

State	Irrep.	Nature	Osc. Str. / a.u.	ΔE / eV
Q_0	B	G.S.	--- [---]	0.00 [0.00]
Q_1	A	$(d_{z2}/d_{xy}) \rightarrow (d_{xy}/d_{yz})$	1.33E-1 [1.50E-1]	1.04 [1.17]
Q_2	A	$(d_{xy}/d_{z2}) \rightarrow (d_{yz}/d_{xy})$	4.65E-5 [5.66E-5]	1.99 [2.42]
Q_3	B	$(d_{yz}/d_{xz}) \rightarrow (d_{xz}/d_{yz})$	2.29E-5 [2.75E-5]	2.19 [2.63]
Q_4	A	$d_{xy} \rightarrow (d_{xz}/d_{yz})$	1.42E-5 [1.72E-5]	2.27 [2.75]

State	Irrep.	Nature	Osc. Str. / a.u.	ΔE / eV
Q_0	B	G.S.	--- [---]	0.23 [0.33]
Q_1	A	$(d_{z}^2/d_{xy}) \rightarrow (d_{xy}/d_{yz})$	1.34E-7 [8.80E-8]	0.24 [0.34]
Q_2	A	$(d_{xy}/d_{z}^2) \rightarrow (d_{yz}/d_{xy})$	1.35E-5 [1.67E-5]	1.82 [2.32]
Q_3	B	$(d_{yz}/d_{xz}) \rightarrow (d_{xz}/d_{yz})$	4.12E-5 [5.12E-5]	1.82 [2.32]
Q_4	A	$d_{xy} \rightarrow (d_{xz}/d_{yz})$	1.60E-8 [2.00E-8]	1.93 [2.47]

Table S2: CASSCF computed excitation energies and state assignments for $Mn(acac)_3$ at C_2 and D_3 point group respectively.

S3.3 Potential energy surface scan details

i	$\Delta E(Q_0)$ / eV	$\Delta E(Q_1)$ / eV	$\Delta E(Q_2)$ / eV	$\Delta E(Q_3)$ / eV	$\Delta E(Q_4)$ / eV
-5	-0.013	1.663	2.610	2.908	3.018
-4	-0.017	1.556	2.563	2.846	2.956
-3	-0.018	1.452	2.521	2.787	2.898
-2	-0.016	1.352	2.482	2.732	2.843
-1	-0.009	1.255	2.448	2.680	2.792
0 (FC)	0.000	1.161	2.417	2.632	2.744
1	0.013	1.070	2.390	2.586	2.700
2	0.029	0.982	2.366	2.544	2.660
3	0.049	0.897	2.347	2.505	2.623
4	0.072	0.815	2.330	2.470	2.589
5	0.098	0.736	2.318	2.437	2.559
6	0.128	0.660	2.308	2.408	2.533
7	0.162	0.588	2.303	2.382	2.511
8	0.199	0.519	2.300	2.359	2.493
9	0.241	0.454	2.302	2.340	2.479
10	0.286	0.393	2.306	2.325	2.471
11 (CI)	0.335	0.335	2.313	2.313	2.467
12	0.282	0.389	2.306	2.324	2.471
13	0.234	0.448	2.302	2.337	2.480
14	0.190	0.512	2.303	2.354	2.498
15	0.152	0.581	2.309	2.373	2.523
16	0.119	0.656	2.320	2.395	2.556
17	0.092	0.737	2.336	2.420	2.598
18	0.072	0.825	2.358	2.451	2.648
19	0.059	0.920	2.386	2.487	2.707
20	0.053	1.022	2.421	2.528	2.774
21	0.055	1.131	2.463	2.576	2.850

Table S3. Computed energies of Q_0–Q_4 along the Jahn–Teller distortion coordinate.

S3.4 Vibrational Frequency Calculations and Mode Assignment

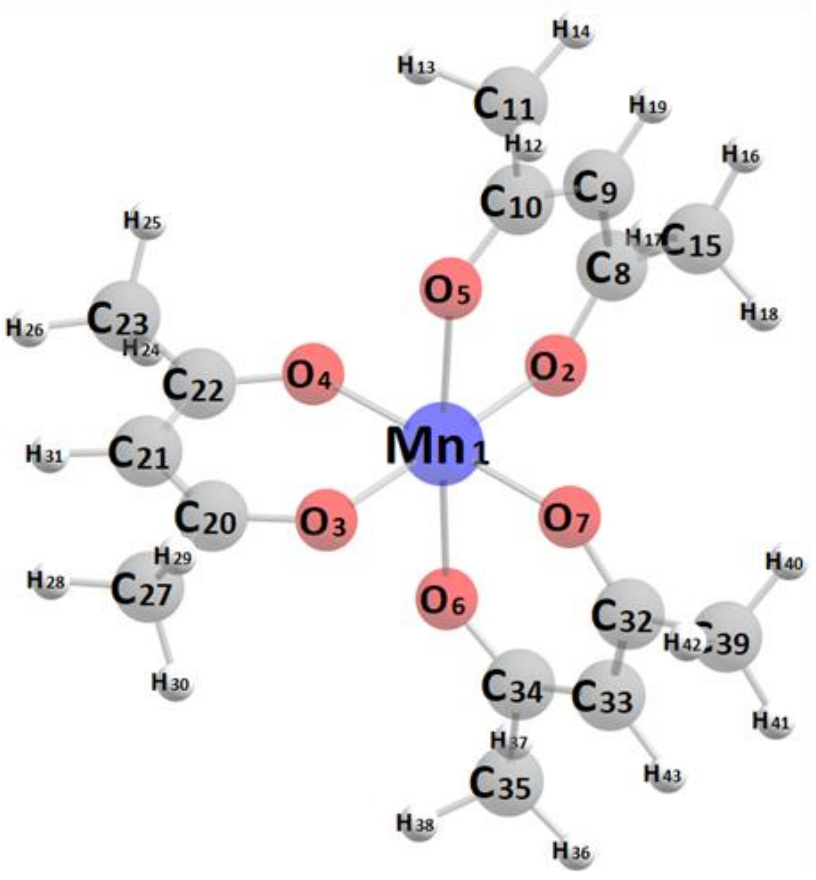

Structural Parameters	Expt.	B3LYP/def2-TZVP	
		Q0Min	CI
Mn(1)-O(5) / Å	2.157	2.172	2.014
Mn(1)-O(6) / Å	2.157	2.172	2.014
Mn(1)-O(2) / Å	1.932	1.937	2.014
Mn(1)-O(3) / Å	1.946	1.951	2.014
Mn(1)-O(4) / Å	1.946	1.951	2.014
Mn(1)-O(7) / Å	1.932	1.937	2.014
∡(O(5)-Mn(1)-O(2) / °	88.9	87.2	88.0
∡(O(7)-Mn(1)-O(6) / °	88.9	87.2	88.0
∡(O(4)-Mn(1)-O(3) / °	91.4	89.6	88.0

Figure S9: Optimised ground-state geometry of Mn(acac)$_3$ showing the elongated JT distortion along the axial Mn–O bonds. Table S4: Selected optimised bond lengths and angles for the elongated ground-state geometry of Mn(acac)$_3$ compared to crystallographic values

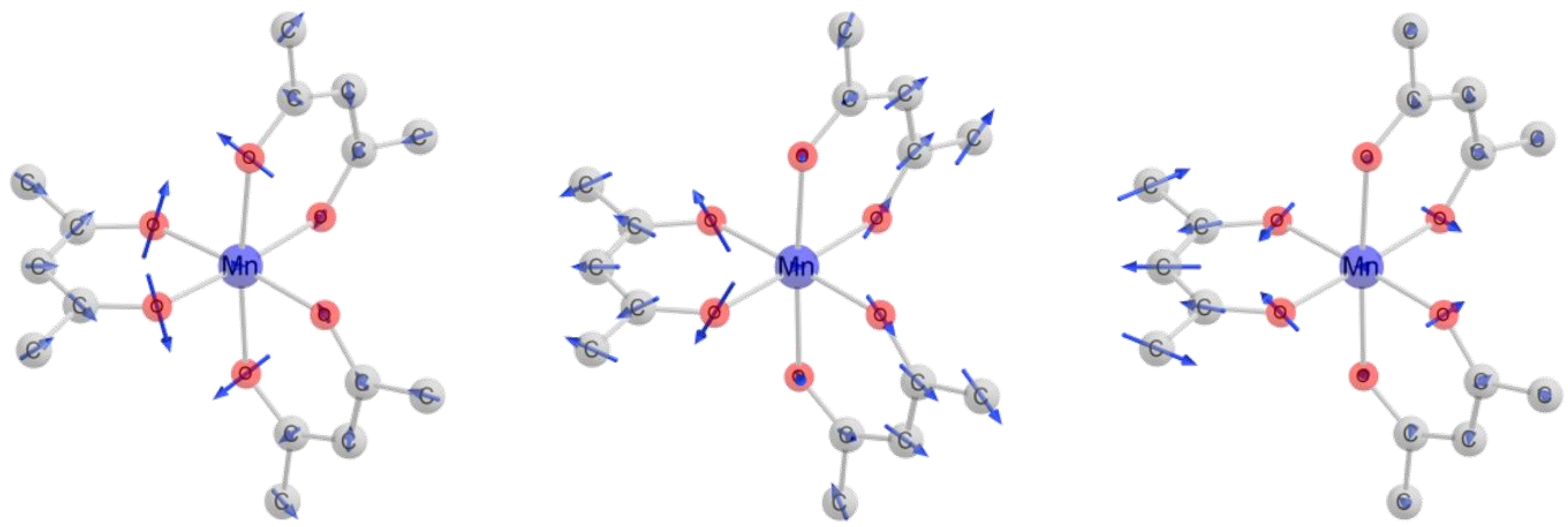

$\omega_{21} = 183.6\ \text{cm}^{-1}$ $\omega_{23} = 198.5\text{cm}^{-1}$ $\omega_{27} = 260.1\ \text{cm}^{-1}$

Figure S10. Normal-mode displacement vectors for the 184, 199, and 260 cm^{-1} modes overlaid on the optimised geometry of Mn(acac)$_3$. H-atoms have been omitted for clarity.

S3.5 Huang–Rhys (HR) factors and mode-resolved reorganisation energy

Mode	Γ	Freq / cm^{-1}	Reorganisation energy / cm^{-1}	Huang-Rhys factor
1	(a)	19.61	0.038 [5.424]	0.002 [0.277]
2	(b)	21.45	0.000 [0.000]	0.000 [0.000]
3	(b)	28.84	0.000 [0.000]	0.000 [0.000]
4	(a)	40.32	0.007 [8.326]	0.000 [0.207]
5	(b)	44.18	0.000 [0.000]	0.000 [0.000]
6	(a)	45.37	0.015 [1.331]	0.000 [0.029]
7	(b)	62.36	0.000 [0.000]	0.000 [0.000]
8	(a)	62.6	0.003 [0.143]	0.000 [0.002]

9	(b)	81.56	0.000 [0.000]	0.000 [0.000]
10	(a)	91.98	0.000 [0.110]	0.000 [0.001]
11	(a)	107.24	0.016 [0.002]	0.000 [0.000]
12	(b)	107.47	0.000 [0.000]	0.000 [0.000]
13	(a)	133.24	0.427 [5.776]	0.003 [0.043]
14	(b)	138.9	0.000 [0.000]	0.000 [0.000]
15	(b)	156.4	0.000 [0.000]	0.000 [0.000]
16	(b)	159.63	0.001 [0.002]	0.000 [0.000]
17	(a)	159.67	152.602 [447.446]	0.956 [2.802]
18	(a)	163.87	4.286 [10.320]	0.026 [0.063]
19	(b)	164	0.000 [0.000]	0.000 [0.000]
20	(b)	169.47	0.000 [0.000]	0.000 [0.000]
21	(a)	183.6	147.214 [408.892]	0.802 [2.227]
22	(b)	189.71	0.000 [0.000]	0.000 [0.000]
23	(a)	198.55	222.411 [474.487]	1.120 [2.390]
24	(a)	233.37	6.035 [5.399]	0.026 [0.023]
25	(b)	234.08	0.000 [0.000]	0.000 [0.000]
26	(a)	248.32	12.058 [28.946]	0.049 [0.117]
27	(a)	260.11	7.759 [6.672]	0.030 [0.026]
28	(b)	269.04	0.000 [0.000]	0.000 [0.000]
29	(b)	331.42	0.000 [0.000]	0.000 [0.000]
30	(a)	334.09	3.406 [3.612]	0.010 [0.011]
31	(a)	408.61	84.799 [52.063]	0.208 [0.127]
32	(b)	410.56	0.000 [0.000]	0.000 [0.000]
33	(b)	414.5	0.000 [0.000]	0.000 [0.000]
34	(b)	427.09	0.000 [0.000]	0.000 [0.000]
35	(a)	438.65	24.481 [15.564]	0.056 [0.035]
36	(a)	453.24	138.909 [69.838]	0.306 [0.154]
37	(b)	554.3	0.000 [0.000]	0.000 [0.000]
38	(a)	562.52	138.391 [27.640]	0.246 [0.049]
39	(a)	571.53	0.538 [0.067]	0.001 [0.000]
40	(b)	572.87	0.000 [0.000]	0.000 [0.000]
41	(a)	574.66	2.416 [0.443]	0.004 [0.001]
42	(b)	593.43	0.000 [0.000]	0.000 [0.000]
43	(b)	663.38	0.000 [0.000]	0.000 [0.000]
44	(a)	667.92	1.223 [15.987]	0.002 [0.024]
45	(b)	676.01	0.000 [0.000]	0.000 [0.000]
46	(a)	676.96	2.501 [0.313]	0.004 [0.000]
47	(a)	677.83	29.575 [15.726]	0.044 [0.023]
48	(b)	684.39	0.000 [0.000]	0.000 [0.000]
49	(b)	789.58	0.000 [0.000]	0.000 [0.000]
50	(a)	789.64	0.003 [0.000]	0.000 [0.000]
51	(b)	792.82	0.000 [0.000]	0.000 [0.000]
52	(b)	937.85	0.000 [0.000]	0.000 [0.000]

53	(a)	939.07	157.348 [3.655]	0.168 [0.004]
54	(b)	946.25	0.000 [0.000]	0.000 [0.000]
55	(b)	953.83	0.000 [0.000]	0.000 [0.000]
56	(a)	955.16	54.801 [15.507]	0.057 [0.016]
57	(a)	968.42	0.797 [4.224]	0.001 [0.004]
58	(b)	1035.59	0.007 [0.000]	0.000 [0.000]
59	(a)	1036.18	31.705 [0.906]	0.031 [0.001]
60	(b)	1039.01	0.001 [0.000]	0.000 [0.000]
61	(b)	1041.84	0.001 [0.000]	0.000 [0.000]
62	(a)	1043.69	33.619 [18.023]	0.032 [0.017]
63	(b)	1044.28	0.001 [0.000]	0.000 [0.000]
64	(a)	1044.51	0.004 [0.196]	0.000 [0.000]
65	(b)	1047.45	0.000 [0.000]	0.000 [0.000]
66	(a)	1051.17	42.412 [8.675]	0.040 [0.008]
67	(b)	1058.41	0.000 [0.000]	0.000 [0.000]
68	(a)	1058.76	0.013 [0.826]	0.000 [0.001]
69	(a)	1059.87	0.001 [0.001]	0.000 [0.000]
70	(b)	1217.5	0.000 [0.000]	0.000 [0.000]
71	(a)	1221.88	34.776 [1.510]	0.028 [0.001]
72	(b)	1222.27	0.002 [0.000]	0.000 [0.000]
73	(b)	1273.71	0.001 [0.000]	0.000 [0.000]
74	(a)	1278.72	51.619 [8.566]	0.040 [0.007]
75	(a)	1300.5	0.896 [0.217]	0.001 [0.000]
76	(b)	1390.59	0.005 [0.000]	0.000 [0.000]
77	(a)	1390.66	1.985 [0.101]	0.001 [0.000]
78	(b)	1394.22	0.000 [0.000]	0.000 [0.000]
79	(b)	1396.22	0.002 [0.000]	0.000 [0.000]
80	(a)	1397	0.198 [0.017]	0.000 [0.000]
81	(a)	1397.19	0.013 [0.001]	0.000 [0.000]
82	(b)	1400.24	0.001 [0.000]	0.000 [0.000]
83	(a)	1432.14	231.863 [56.476]	0.162 [0.039]
84	(b)	1436.24	0.005 [0.000]	0.000 [0.000]
85	(a)	1471.5	0.001 [0.026]	0.000 [0.000]
86	(b)	1471.54	0.000 [0.000]	0.000 [0.000]
87	(a)	1471.68	0.020 [0.057]	0.000 [0.000]
88	(b)	1472.16	0.000 [0.000]	0.000 [0.000]
89	(a)	1473.64	0.020 [0.042]	0.000 [0.000]
90	(b)	1473.66	0.000 [0.000]	0.000 [0.000]
91	(b)	1475.9	0.002 [0.000]	0.000 [0.000]
92	(a)	1476.58	0.635 [0.100]	0.000 [0.000]
93	(a)	1477.41	2.947 [0.193]	0.002 [0.000]
94	(b)	1486.05	0.000 [0.000]	0.000 [0.000]
95	(b)	1493.71	0.000 [0.000]	0.000 [0.000]
96	(a)	1495.73	81.691 [23.553]	0.055 [0.016]

97	(b)	1543.53	0.000 [0.000]	0.000 [0.000]
98	(a)	1544.5	4.574 [3.810]	0.003 [0.002]
99	(b)	1556.05	0.000 [0.000]	0.000 [0.000]
100	(a)	1609.11	63.533 [7.644]	0.039 [0.005]
101	(b)	1630.09	0.000 [0.000]	0.000 [0.000]
102	(a)	1649.1	2.552 [0.824]	0.002 [0.000]

Table S5. Mode-resolved Huang–Rhys factors and reorganisation-energy contributions for Q_1 excitation of $Mn(acac)_3$. Values given in square brackets are values estimated by differentiation between values at the conical intersection and Franck-Condon geometries. The value in square brackets for Huang-Rhys factors are computed as half of the displacement between the CI and FC geometries along each normal mode. High frequency modes (> 3000 cm^{-1}) corresponding to H motions are omitted. Γ is the irreducible representation of the normal mode in the C_2 point group symmetry.

S4. Full Transient Absorption Dataset

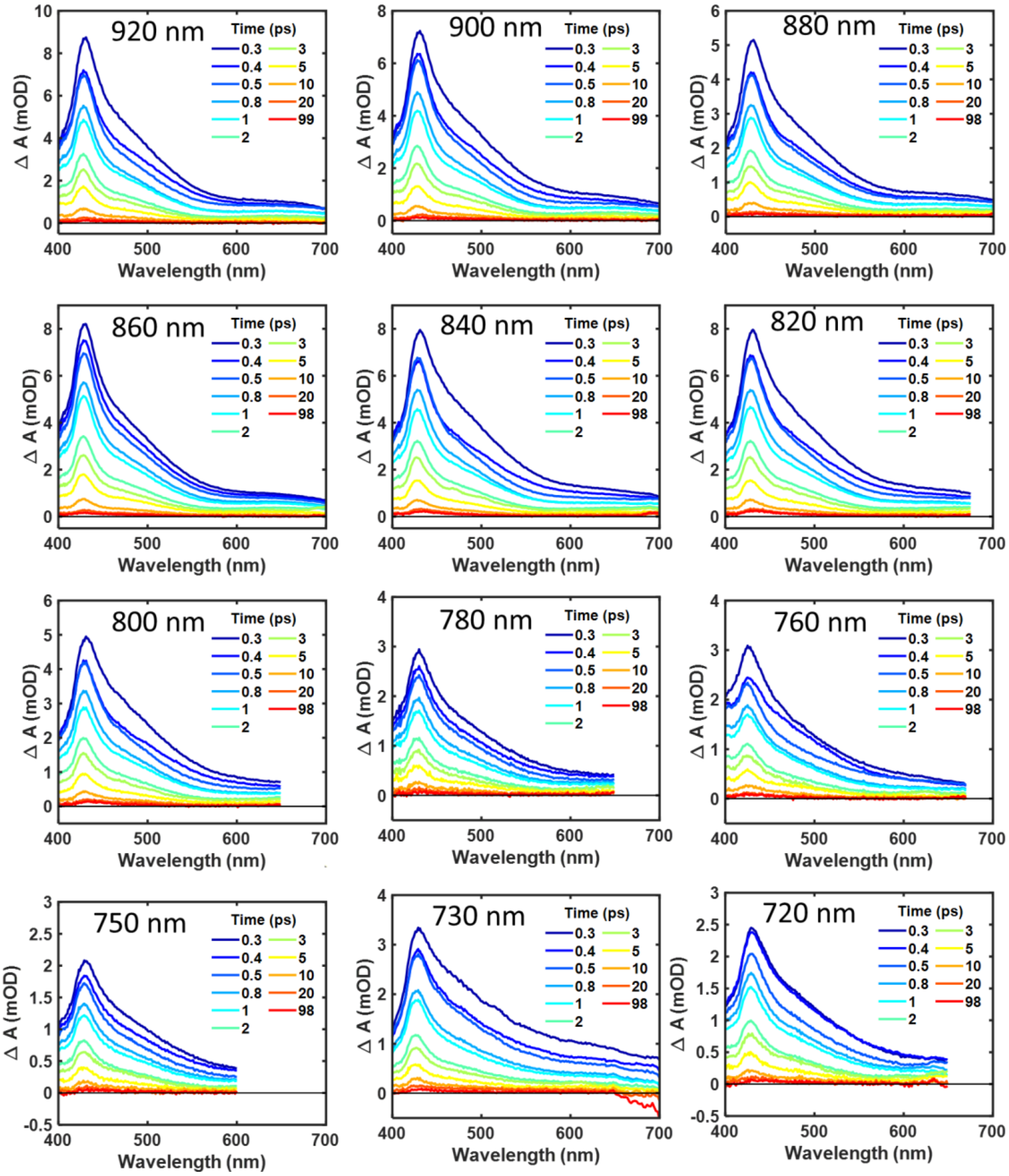

Figure S11. TA spectra of $Mn(acac)_3$ in ethanol for Q_1-region excitation wavelengths.

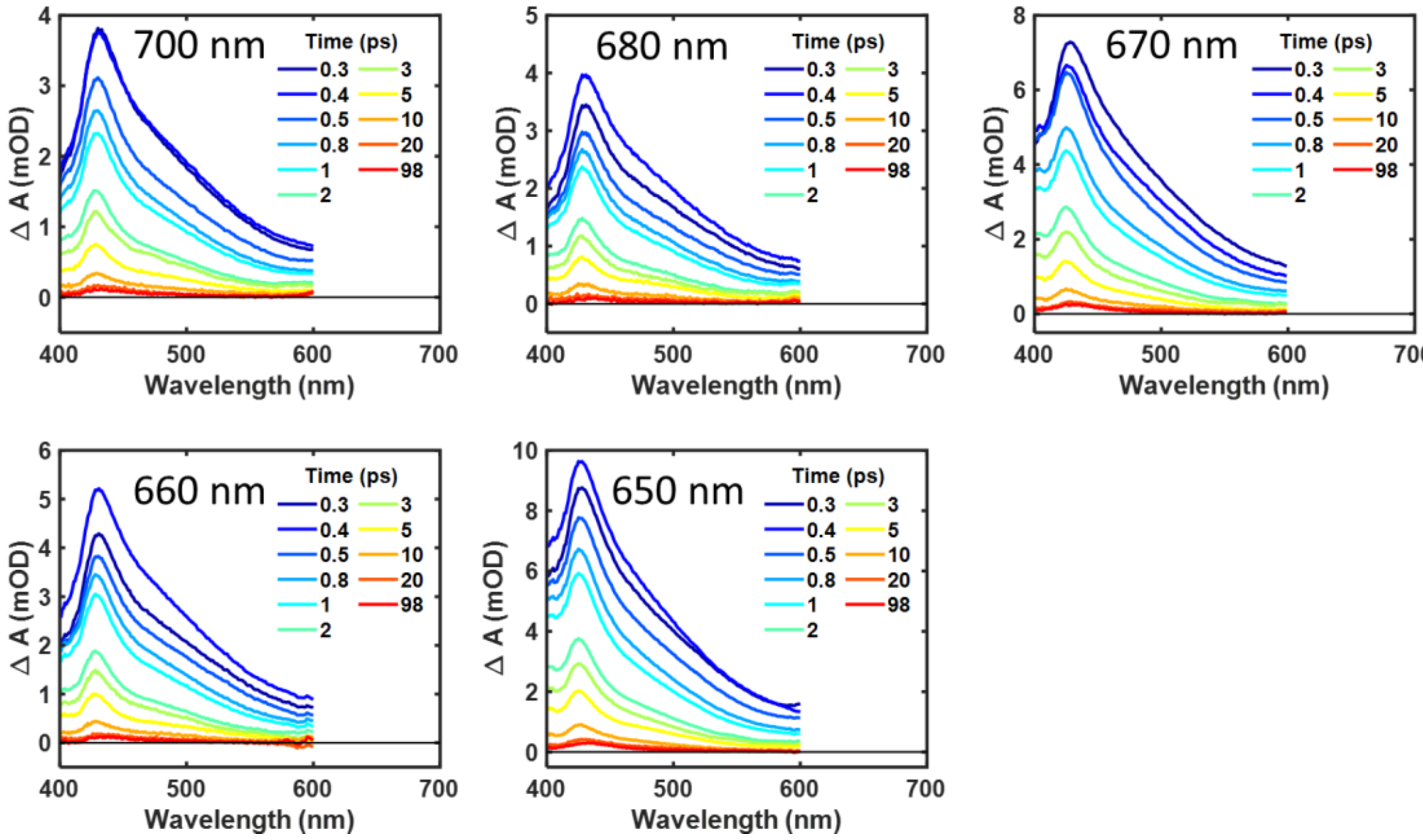

Figure S12. TA spectra of $Mn(acac)_3$ in ethanol for Q_2-region excitation wavelengths.

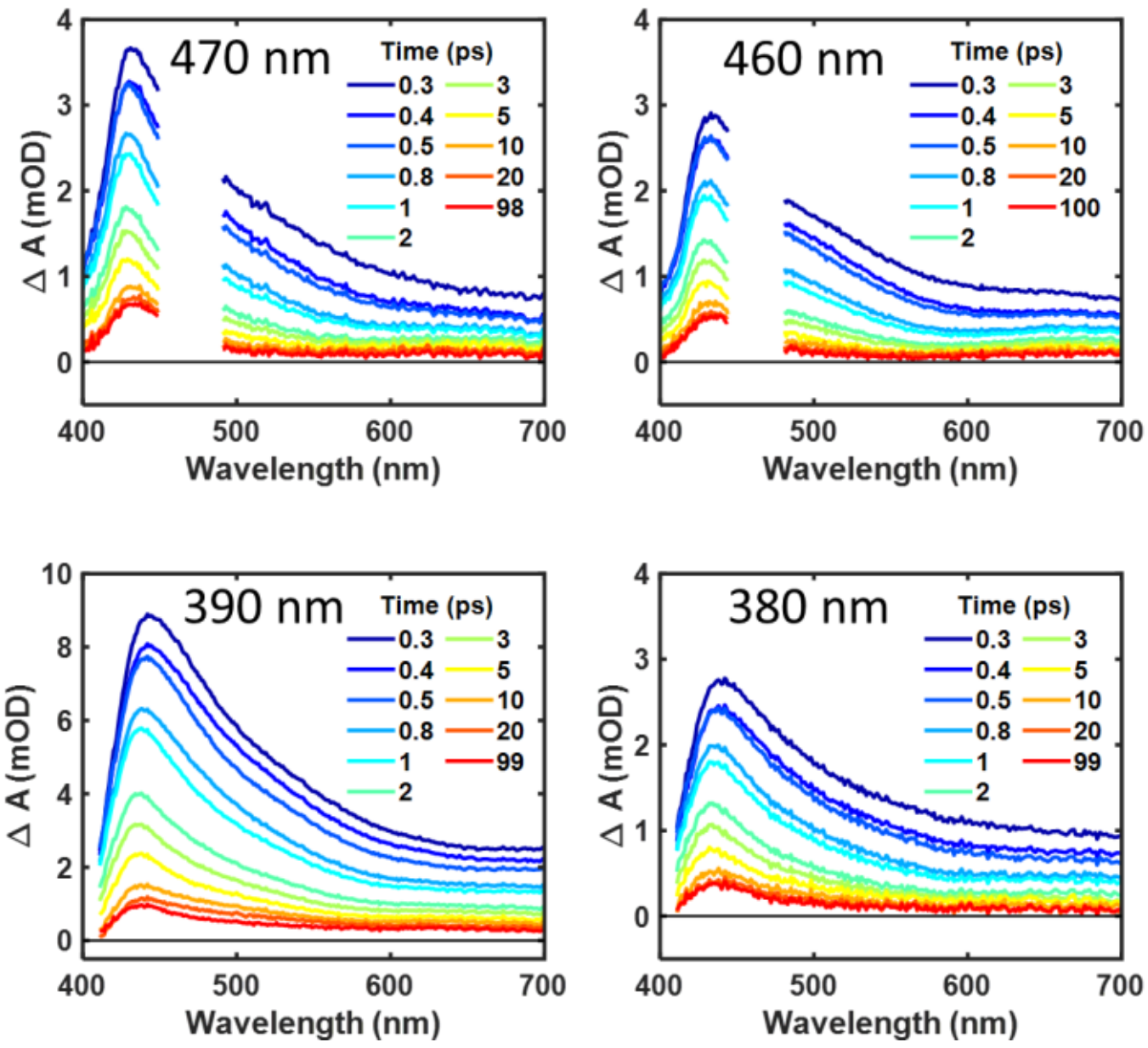

Figure S13. TA spectra of $Mn(acac)_3$ in ethanol for $Q_{3,4}$-region excitation wavelengths.

S5. Spectral Shift of the TAS Feature

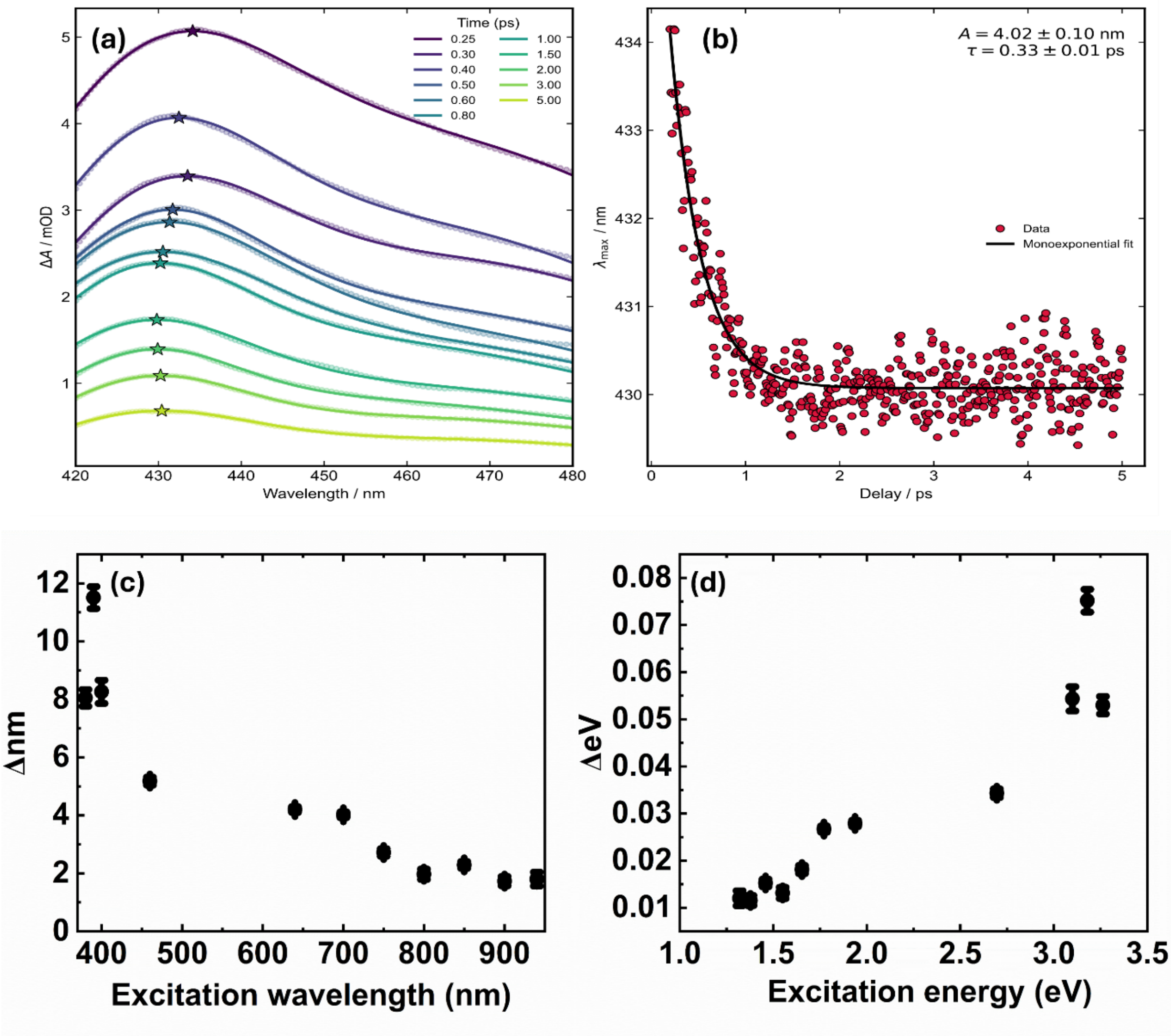

Figure S14: Quantification of the excitation-wavelength dependence of the transient absorption (TA) spectral shift. (a) Representative TA spectra taken at 640-nm excitation and corresponding fits obtained using the sum of two Gaussian functions at each pump-probe delay. (b) Peak positions extracted from the fitted spectra as a function of delay time. The temporal evolution of the peak position was fitted with a single-exponential function, and the magnitude of the spectral shift, Δν, was extracted from the fit amplitude. Uncertainties were obtained directly from the errors associated with the exponential fits. These values are likely underestimated, as the determined peak positions are influenced not only by the quality of the fit but also by the presence of oscillatory features in the data. (c) Spectral shift, Δν, as a function of excitation wavelength. (d) Spectral shift, Δν, expressed in energy units.

S6. Time-Window Sensitivity of the Relative compressed state yield

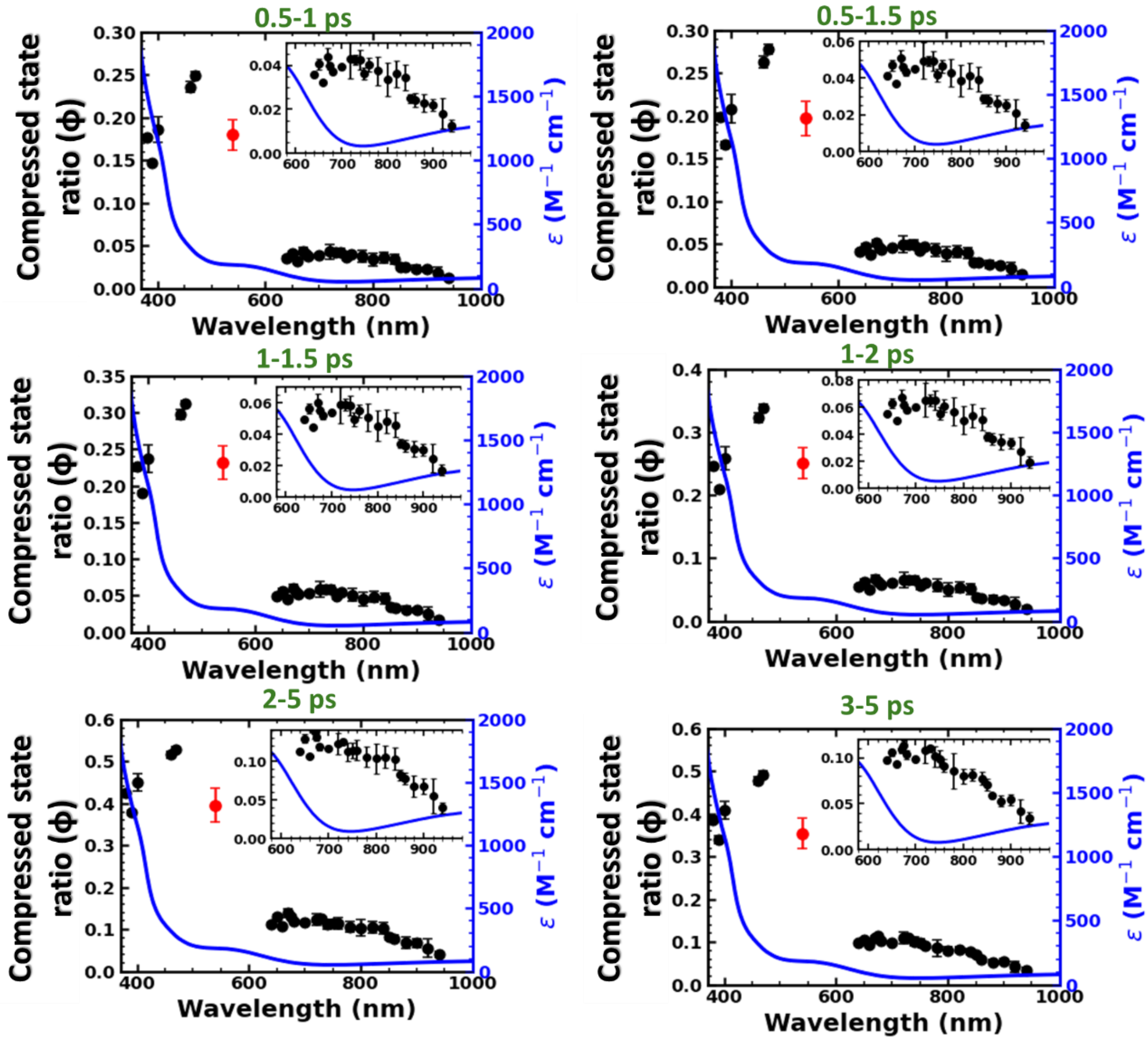

Figure S15. Compressed-state ratio as a function of excitation wavelength for different early- and late-time averaging windows.

S7. Landau–Zener Model and Derivation of Equation 3

A fraction, A, of the total excess energy, ($E_{\text{excess}} = E_{\text{Excitation}} - E_{\text{CI}}$), is deposited into the reactive Jahn–Teller (JT) coordinate, such that the energy available along this coordinate is given by

$$E_{\text{rc}} = A(E_{\text{Excitation}} - E_{\text{CI}})$$

Increasing the excitation energy therefore increases the kinetic energy (E_k) and velocity (v) of the nuclear wavepacket approaching the conical intersection. Since the wavepacket velocity is proportional to the square root of the kinetic energy at the crossing,

$$v = \sqrt{\frac{2E_k}{\mu}} = \sqrt{\frac{2A(E_{\text{Excitation}} - E_{\text{CI}})}{\mu}} = \sqrt{\frac{2E_{\text{rc}}}{\mu}}$$

where μ is the reduced mass and E_{CI} is the energy of the conical intersection, the Landau–Zener (LZ) probability for the non-adiabatic transition can be rewritten as

$$P = exp\left(\frac{-\sqrt{2\mu}\pi V_{12}^2}{\hbar\sqrt{E_{\mathrm{rc}}}|\Delta F|}\right)$$

where V_{12} is the electronic coupling between the diabatic states and ΔF is the difference in slopes of the intersecting potential energy surfaces at the crossing. The experimentally observed relative compressed-state ratio (φ) is assumed to be directly proportional to this transition probability, such that

$$\varphi = B.P$$

where B is a scaling factor accounting for the extinction coefficients of the excited state and compressed ground state. Substituting the LZ expression for (P) gives

$$\varphi(E_{\mathrm{Excitation}}) = Bexp\left(\frac{-C}{\sqrt{E_{\mathrm{rc}}}}\right)$$

where C consolidates the constant theoretical parameters $(\mu, V_{12}, \Delta F)$. The quantities E_{CI} and A can be estimated from quantum chemical calculations.

1. F, Neese, Software update: the ORCA program system, version 6.0, *WIRES Comput. Molec. Sci.,* 2025 **15(1)**, e70019

2. P. J. Stephens, F. J. Devlin, C. F. Chabalowski, and M. J. Frisch., *J. Phys. Chem.,* 1994 **98**, 11623

3. F. Weigend, Accurate Coulomb-fitting Basis Sets for H to Rn., *Phys. Chem. Chem. Phys.,* 2006 **8**, 1057−1065

4.G. Jansen and B. A. Hess, *Physical Review A*, 1989 **39**, 6016–6017.

5.B. A. Hess, *Physical Review A*, 1986 **33**, 3742–3748.

6.B. A. Hess, *Physical Review A*, 1985 **32**, 756–763.

7. M. Douglas and N. M. Kroll, *Annals of Physics*, 1974 **82**, 89–155

8. C. Kollmar, K. Sivalingam, B. Helmich-Paris, C. Angeli, F. Neese, A perturbation-based super-CI approach for the orbital optimization of a CASSCF wave function. *J. Comput. Chem.*, 2019 **40 (14)**, 1463–1470

9. T.J. Penfold, J. Eng, Mind the GAP: Quantifying the Breakdown of the Linear Vibronic Coupling Hamiltonian. *Phys. Chem. Chem. Phys.,* 2023 **25 (10)**, 7195−7204.
